# Discovery of *Alanomyces manoharacharyi*: A Novel Fungus Identified Using Genome Sequencing and Metabolomic Analysis

**DOI:** 10.3390/jof10110791

**Published:** 2024-11-14

**Authors:** Shiwali Rana, Sanjay K. Singh

**Affiliations:** National Fungal Culture Collection of India, Biodiversity and Palaeobiology Group, MACS’ Agharkar Research Institute, GG Agarkar Road, Pune 411004, India

**Keywords:** AntiSMASH, asexual morph, endophyte, genome mining, MALDI-TOF, metabolite profiling, novel taxon, phylogeny

## Abstract

In this study, a new species of *Alanomyces* was isolated as an endophyte from the bark of *Azadirachta indica* from Mulshi, Maharashtra. The identity of this isolate was confirmed based on the asexual morphological characteristics as well as multi-gene phylogeny based on the internal transcribed spacer (ITS) and large subunit (LSU) nuclear ribosomal RNA (rRNA) regions. As this was the second species to be reported in this genus, we sequenced the genome of this species to increase our knowledge about the possible applicability of this genus to various industries. Its genome length was found to be 35.01 Mb, harboring 7870 protein-coding genes as per Augustus and 8101 genes using GeMoMa. Many genes were annotated using the Clusters of Orthologous Groups (COGs) database, the Kyoto Encyclopedia of Genes and Genomes (KEGG), Gene Ontology (GO), Swiss-Prot, NCBI non-redundant nucleotide sequences (NTs), and NCBI non-redundant protein sequences (NRs). The number of repeating sequences was predicted using Proteinmask and RepeatMasker; tRNA were detected using tRNAscan and snRNA were predicted using rfam_scan. The genome was also annotated using the Pathogen–Host Interactions Database (PHI-base) and AntiSMASH. To confirm the evolutionary history, average nucleotide identity (ANIb), phylogeny based on orthologous proteins, and single nucleotide polymorphisms (SNPs) were carried out. Metabolic profiling of the methanolic extract of dried biomass and ethyl acetate extract of the filtrate revealed a variety of compounds of great importance in the pharmaceutical and cosmetic industry. The characterization and genomic analysis of the newly discovered species *Alanomyces manoharacharyi* highlights its potential applicability across multiple industries, particularly in pharmaceuticals and cosmetics due to its diverse secondary metabolites and unique genetic features it possesses.

## 1. Introduction

The potential of fungal endophytes to produce bioactive substances that can be utilized in a variety of applications, such as agriculture, medicine, and biocontrol methods, has attracted a significant amount of attention. Fungal endophytes are organisms that live within the interior tissues of plants. The neem plant, *Azadirachta indica*, is a model that is particularly useful for the investigation of these endophytes. Several studies have documented the presence of several fungal taxa throughout diverse plant parts, including leaves, stems, and bark [1,2]. Based on these findings, research has demonstrated that the diversity of fungal endophytes associated with neem is extensive.

Verma et al. [2] carried out a comprehensive investigation that resulted in the identification of 233 different endophytic fungi from neem, with most of these fungi being of the Hyphomycetes species. According to Sharma et al. [3], this diversity is extremely important since different endophytes have the potential to produce different secondary metabolites, each of which can have a unique set of biological activities. According to Umurhurhu et al. [4], the capacity of endophytes to produce a diverse array of compounds is determined by a number of factors, including the species of the plant that serves as their host, the conditions of the surrounding environment, and the particular interactions that occur between the endophytes and their host.

According to the findings of the research conducted on fungal endophytes derived from neem, these endophytes have the potential to be used in biological control, notably against phytopathogens. Nthuku et al. [1] demonstrated that fungal endophytes isolated from neem had high biocontrol potential against *Fusarium oxysporum* f.sp. *cubense*, which is a pathogen responsible for producing *Fusarium* wilt in bananas. The findings of this study demonstrate the significance of endophytes in improving the health of plants and developing their resistance to illnesses. It is also important to highlight the wide variety of bioactive substances that endophytic fungi create in terms of their chemical composition. For instance, Fulzele’s research on *Preussia isabellae*, an endophyte that was isolated from neem, found that it possesses the ability to create lovastatin, a chemical that possesses strong antibacterial characteristics [5]. In a similar way, Chukwuemerie et al. highlighted the antimalarial potentials of secondary metabolites obtained from *Curvularia lunata*, which is another endophyte that originates from neem. This highlights the medicinal potential of these fungi [6]. Based on this evidence, it appears that the endophytic community that is associated with neem not only makes an impact on the overall health of the plant, but it also acts as a reservoir for the discovery of new bioactive chemicals.

Endophytic fungi that are produced from neem have a wide range of potential applications, particularly in the field of sustainable agriculture. The application of these fungi as biocontrol agents has the potential to reduce the reliance on synthetic pesticides, hence supporting practices that are more environmentally friendly. Studies have shown that endophytic fungi have the ability to successfully restrict the growth of agricultural pests and pathogens, which in turn leads to an increase in crop yield and quality [1,4]. Furthermore, the investigation of endophytes for their antimicrobial properties has significance for the development of new medications. This is because many endophytes produce compounds that have antibacterial, antifungal, and antiviral activity [7].

Keeping in view the rich diversity of endophytes associated with neem, coupled with their ability to produce a wide array of bioactive compounds, positions them as valuable resources for biocontrol and therapeutic applications; we tried isolating various fungi associated with neem and resulted in the isolation of an interesting new species of *Alanomyces*. The genus *Alanomyces* belongs to the *Aplosporellaceae* Slippers, Boissin, and Crous family [8]. This family, *Aplosporellaceae*, was erected in 2013 under the order *Botryosphaeriales*. *Aplosporella* was the genus included in this family at the time of its establishment. The type genus was *Aplosporella* Speg. and the type species was *Aplosporella chlorostroma* Speg. [9]. Later, in 2017, another genus, *Alanomyces*, was established and included in this family. The type species was *Alanomyces indica*. This is the only species reported to date in this genus; it was isolated from soil attached to the fruiting body of an unidentified macrofungus from India [10]. Many species of *Aplosporella* have been associated with thin, dead twigs and rarely occur on leaves or thicker branches [11]. Species of *Aplosporella* have been reported from *Acacia cochlearis*, *Acacia erioloba*, *Acacia mellifera*, *Acacia tortillas*, *Acer buergerianum*, *Artocarpus heterophyllus*, *Celtis africana*, *Cerasus yedoensis*, *Cercis chinensis* f. *chinensis*, *Chaenomeles sinensis*, *Eucalyptus gomphocephala*, *Gleditsia sinensis*, *Juglans regia*, *Juniperus chinensis*, *Mimetes cucullate*, *Prunus persica* var. *nucipersica*, *Searsia lancea*, *Sophora japonica*, *Ziziphus jujuba*, etc. [12]. In this study, we describe a new species belonging to the genus *Alanomyces* isolated as an endophyte from the bark of *Azadirachta indica* from Mulshi, Maharashtra. Also, we present here the genomic and metabolic profiling of this novel species, which can help improve its applicability for the benefit of humanity.

## 2. Materials and Methods

### 2.1. Collection, Isolation, and Morphological Characterization

The bark of *Azadirachta indica* was collected from Mulshi, Maharashtra, India, on 11 February 2024 and was placed in sterile polythene bags and transported carefully to the laboratory. The surface adherents were removed after thoroughly washing them under tap water. Then, larger pieces were chopped into smaller ones and subjected to surface sterilization following a modified method by Dobranic et al. [13]. Concisely, the bark of *Azadirachta indica* was first dipped in ethanol (70%) for 5 s, followed by sodium hypochlorite (4%) for 90 s, and later rinsed with sterile water for 10 s (four times). These surface sterilized pieces were cut into small pieces using a sterilized sharp blade No. 10 (Sigma-Aldrich Chemicals Private Ltd., Bangalore, India) and inoculated on potato dextrose agar (PDA) plates. These plates were kept at 25 °C until any vegetative growth appeared from the inoculated tissues. Individual colonies from inoculated tissues were transferred to fresh PDA plates by hyphal tipping and allowed to grow into pure cultures [14]. For further studies, two similar-looking colonies were maintained on PDA. Colony characteristics of this isolate were studied on PDA, malt extract agar (MEA), V8 juice agar, corn meal agar (CMA), rose bengal agar (RBA), czapek dox agar (CDA), potato carrot agar (PCA), and sabouraud dextrose agar (SDA). Methuen’s Handbook of Color was referred to for recording the colors of the colonies on different agar media [15]. Microscopic structures of the isolates were recorded from pure culture using a staining cum-mounting medium, lactophenol cotton blue, under a Carl Zeiss Image Analyzer 2 (Oberkochen, Germany) microscope. Measurements and photomicrographs of the fungal structures were recorded using Axiovision Rel 4.8 software and Digi-Cam attached with the Carl Zeiss Image Analyzer 2 microscope. The holotype specimen was deposited and accessioned in the Ajrekar Mycological Herbarium (AMH 10702) and ex-type pure culture was deposited and accessioned in the National Fungal Culture Collection of India (NFCCI 5738).

### 2.2. DNA Extraction, PCR Amplification, and DNA Sequencing

Genomic DNA was extracted from pure colonies raised from single spore isolation on PDA Petri plates. After approximately one week of incubation, DNA extraction was performed using a simple, easy, and rapid DNA extraction protocol using the FastPrep^®^24 tissue homogenizer (MP Biomedicals GmbH, Eschwege, Germany) [16]. The amplification and sequencing of ITS and LSU gene regions were carried out. The primers involved in amplification and sequencing were ITS-5 (5′ GGAAGTAAAAGTCG-TAACAAGG 3′) and ITS-4 (5′ TCCTCCGCTTATTGATATGC 3′) for ITS [17] and LR-0R (5′ ACCCGCTGAACTTAAGC 3′) [18] and LR-7 (5′ TACTACCACCAAGATCT 3′ [19] for the 28S large subunit of the nrDNA (LSU). A 25 μL reaction was used to perform the PCR using 12.5 μL 2× Invitrogen Platinum SuperFi PCR Mastermix, 2 μL template DNA (10–20 ng), 1.5 μL 10 pmol primer, 5 μL 5× GC enhancer, and H_2_O (Sterile Ultra-Pure Water, Sigma-Aldrich, St. Louis, MI, USA), with the total volume made to equal 25 μL. The conditions of the thermocycling involved those as follows: for the ITS gene region, an initial denaturation at 94 °C for 5 min, 35 cycles of 1 min at 94 °C, 30 s at 52 °C, 1 min at 72 °C, and lastly an extension at 72 °C for 8 min; for LSU, 5 min denaturation at 95 °C, 35 cycles of 60 s at 95 °C, 50 s at 52 °C, and 1.2 min at 72 °C, with a final 8 min extension at 72 °C. Unfortunately, after repeated trials to amplify the LSU gene region, we failed; therefore, the sequence of the LSU gene region was collected from the whole-genome sequencing data (details of sequencing, assembly, and annotation are mentioned subsequently in this paper). Per the manufacturer’s instructions, the PCR amplicons were purified with a FavorPrep™ PCR Purification Kit (Favorgen Biotech Corp., Ping Tung, Taiwan). Purified PCR products of both marker genes were checked on 1.2% agarose gel electrophoresis stained with 0.5 μg/mL ethidium bromide. They were further subjected to a sequencing PCR using a BigDye^®^Terminator v3.1 Cycle Sequencing Kit per the manufacturer’s instructions. In brief, the sequencing PCR of 20 μL included 4 μL 5× sequencing buffer, 2 μL BigDye™ Terminator premix, 4 μL primer (5 pmol), and 4 μL purified amplicon and H_2_O (Sterile Ultra-Pure Water, Sigma), with the volume equaling 20 μL. Thermal cycling conditions consisted of denaturation at 96 °C for 3 min, 30 cycles of 94 °C for 10 s, 50 °C for 40 s, and 60 °C for 4 min were performed. The BigDye^®^ terminators and salts were removed using the BigDye Xterminator^®^ Purification Kit (Thermo Fisher Scientific, Waltham, MA, USA) per the manufacturer’s instructions. After performing cycle sequencing with the BigDye™ terminator, 80 μL SAM™ solution and 20 μL XTerminator™ solution were added to each tube. The mixture was vortexed for 30 min and then centrifuged at 10,000 rpm for 30 s. After transferring the supernatant to a 96-well microplate, the module was selected and the run was set up. The sequence was elucidated using the Applied Biosystems SeqStudio Genetic Analyzer (Applied Biosystems, Foster City, CA, USA). Sequences obtained were submitted to NCBI GenBank.

### 2.3. Phylogenetic Analysis

To determine the phylogenetic status of this novel isolate, *Alanomyces manoharacharyi*, ITS and LSU gene regions were used to compare the present isolate with already known authentic strains. The sequences of the related authentic strains were retrieved from NCBI. Sixty-eight isolates belonging to *Aplosporellaceae* and associated families were used in the phylogenetic analysis and aligned with the sequences of *A. manoharacharyi*. *Fusicladium oleagineum* CBS 113427 and *F. convolvularum* CBS 112706 were selected as the outgroup taxa. The strains used in making phylogenetic trees, their accession numbers, and other related details are presented in Table 1. Each gene region was aligned with the MAFFT v. 7 web server (https://mafft.cbrc.jp/alignment/server/ (accessed on 30 June 2024)) using auto strategy. The scoring matrix for nucleotide sequences was 200PAM/k = 2; the gap opening penalty was set at 1.53; the offset value was set at 0; and a Score of N in nucleotide data, i.e., long stretches of Ns that tend to be gapped, were excluded from the alignment [20]. The alignments were checked and adjusted manually using AliView v1.28 [21]. Furthermore, the alignments were concatenated and processed for phylogenetic analyses. The best substitution model was figured using jModelTest v2.1.10 [22]. Furthermore, the phylogenetic tree was generated using the Windows version of the IQ-tree v.1.6.11 [23]. It was determined and tested whether the tree branches were reliable based on 1000 ultrafast bootstrap support replicates (UFBoot) and the SH-like approximate likelihood ratio test (SH-like aLRT) with 1000 replicates. The constructed phylogenetic tree was visualized in FigTree v.1.4.4.

### 2.4. Identification of the Isolate by MALDI-TOF Mass Spectrometry

The present isolate was inoculated in 3 mL sabouraud dextrose broth in a 15 mL Eppendorf conical tube for 24 h at 28 °C in a rotator to shake overhead at 80 rpm. The tubes were removed and placed on a bench for 10 min. Then, 1.5 mL from the sedimented liquid culture was used to prepare the sample for MALDI-TOF MS measurements. The mycelium was pelleted by centrifugation at 13,000 rpm for 2 min, after which the supernatant was removed. The pellet was again dissolved in 1 mL HPLC water and vortexed. The mycelium was pelleted by centrifugation at 13,000 rpm for 2 min, after which the supernatant was removed. Once again, 1 mL of HPLC water was used to dissolve the pellet. The mycelium was vortexed with HPLC water, centrifuged for two minutes at 13,000 rpm, and the supernatant was discarded. Later, 300 μL HPLC water and 900 μL ethanol were added and vortexed. Later, the supernatant was discarded, and the pellet was completely dried after centrifugation at 13,000 rpm for 2 min. Then, 40 μL formic acid and 40 μL acetonitrile were added and mixed carefully. Later, it was centrifuged at full speed for 2 min. Afterward, 1 µL of the supernatant was pipetted onto the MALDI target, overlaid with 1 µL of HCCA matrix, and analyzed with MALDI-TOF. This preparation was placed in three sample positions on a MALDI Biotarget plate. The resulting spectra were assessed using the Bruker Filamentous Fungi Library 3.0.

### 2.5. High Molecular Weight DNA Extraction for Whole-Genome Sequencing

DNA extraction of *Alanomyces manoharacharyi* NFCCI 5738 was carried out using the MasterPure™ Complete DNA and RNA Purification Kit (Cat #MC85200) (LGC Biosearch Technologies, Hoddesdon, UK). Fungal biomass was added to a PowerBead Pro tube (Qiagen, Hilden, Germany). Later, 150 μL tissue and cell lysis solution was added, secured in a TissueLyser (Qiagen, Hilden, Germany), and processed at maximum speed for 0.5–1 min. Subsequently, 1 μL Proteinase K and 150 μL tissue and cell lysate solution were introduced, thoroughly mixed, and incubated at 65 °C for 15 min. After cooling the sample to 37 °C, 1 μL of 5 μg/μL RNase A was added. After the mixture was well mixed, the sample was incubated at 37 °C for 30 min. The sample was placed on ice for 3–5 min; later, 175 μL MPC protein precipitation reagent was added to the lysed sample and mixed well. Centrifugation for 10 min was used to pellet the debris at 4 °C and ≥10,000× *g*. After that, the supernatant was moved to a fresh microcentrifuge tube, to which 500 μL isopropanol was added and thoroughly mixed. Centrifugation for ten minutes was used to pellet the DNA at 4 °C. After discarding the supernatant, the pellet was twice washed with 70% ethanol. DNA was resuspended in TE Buffer. The integrity was evaluated by 1% agarose gel electrophoresis and purity was accessed by a NanoDrop™ 1000 Spectrophotometer (Thermo Fisher Scientific).

### 2.6. Library Preparation and Sequencing

DNA fragmentation and library construction were conducted using the NEBNext^®^ Ultra™ II FS DNA Library Prep Kit (New England Biolabs, Ipswich, MA, USA) for Illumina protocol (Cat #E7805) per the manufacturer’s instructions. DNA fragmentation, end repair, and dA-tailing were carried out in a 0.2 mL-thin-wall PCR tube (Sigma-Aldrich, St. Louis, MI, USA) with 250 ng genomic DNA, 7 µL of NEBNext Ultra II FS Reaction Buffer, and 2 µL of NEBNext Ultra II FS Enzyme Mix. The total reaction volume was made to be 36 µL using nuclease-free water. The incubation was carried out in a thermocycler; conditions involved incubation at 37 °C for 15 min followed by incubation at 65 °C for 30 min. For the ligation of the adaptors to this FS Reaction Mixture, 30 µL of NEBNext Ultra II Ligation Master Mix, 1 µL of NEBNext Ligation Enhancer (New England Biolabs, Ipswich, MA, USA), and 2.5 µL of NEBNext Adaptor for Illumina were added. The thermal cycler was used to incubate this mixture at 20 °C for 15 min. Later, 3 μL USER^®^ enzyme was added to the ligation mixture and incubated at 37 °C for 15 min. NEB-Next sample purification beads were added to clean the adaptor-ligated DNA, which was eluted using 17 μL 0.1× TE. For PCR enrichment of the adaptor-ligated DNA, 15 μL adaptor-ligated DNA fragments, 25 μL NEBNext Ultra II Q5 Master Mix, and 10 μL index/universal primers were mixed and subjected to PCR amplification. The conditions included initial denaturation at 98 °C for 30 s, followed by five cycles of denaturation at 98 °C for 10 s, annealing/extension at 65 °C for 75 s, and a final extension at 65 °C for 5 min. The PCR was cleaned up using 0.9× NEBNext sample purification beads. The DNA was eluted from the beads by adding 33 μL of 0.1× TE. Furthermore, the quality of the library was assessed on tapestation and quantification of the sequencing library was carried out by a Qubit fluorometer (Thermo Fisher Scientific, MA, USA). The libraries were sequenced using Illumina NovaSeq 6000 sequencer v1.5-chemistry (Illumina, San Diego, CA, USA) for 150 bp paired-end sequencing, according to the manufacturer’s procedure.

### 2.7. Genome Assembly

The raw data quality was checked using FastQC v0.12.1 [http://www.bioinformatics.babraham.ac.uk/projects/fastqc/ (accessed on 30 June 2024)] and MultiQC software v.1.23 [24]. The data generated were checked for base call quality distribution, % bases above Q20 and Q30, %GC, and sequencing adapter contamination. The adapter sequence used was the P7 adapter read1 AGATCGGAAGAGCACACGTCTGAACTCCAGTCA and P5 adapter read2 AGATCGGAAGAGCGTCGTGTAGGGAAAGAGTGT. The raw sequence reads were processed using fastp v0.12.4 to remove adapter sequences and low-quality bases [25]. The high-quality reads that passed quality control were subjected to assembly into contigs using three different assemblers: Megahit v1.2.9 [26] with k-mer sizes of 21, 49, 77, 105, 133, and 141; Spades v3.15.4 [27]; and MaSurCa 4.0.5 [28]. Contigs shorter than 1000 base pairs were subsequently removed from the assembly. For evaluation of the quality of the assemblies, the assembled genome statistics were analyzed using QUAST v5.0.2 [29]. The assembly quality was checked by mapping the reads back onto the assembled contigs using bowtie2 v2.4.5 [30]. The genome completeness was checked using BUSCO v5.3.2 with ascomycota_odb10 as a reference [31]. The genome diagram of *A. manoharacharyi* NFCCI 5738 was constructed using Circos version 0.69-9 [32].

### 2.8. Genome Prediction and Annotation

Gene prediction was performed using Augustus 3.4.0 as well as GeMoMa [33,34,35,36]. Based on the gene function and metabolic pathway of the existing databases, the function annotation was performed using BLAST searches against these databases: NRs (NCBI non-redundant protein sequences), NTs (non-redundant nucleotide sequences), Swiss-Prot, the COG (Cluster of Orthologous Groups) of proteins, the KEGG (Kyoto Encyclopedia of Genes and Genomes), GO (Gene Ontology), the PHI-base (Pathogen–Host Interactions Database), CAZy (the Carbohydrate-Active Enzymes Database).

### 2.9. Analysis of Secondary Metabolite Biosynthetic Gene Clusters

Secondary metabolites biosynthetic gene cluster analysis of *Alanomyces manoharacharyi* NFCCI 5738 was carried out by AntiSMASH fungal 7.1.0 [37]. To further study the obtained gene clusters, the NCBI Genome Portal Software Platform (https://www.ncbi.nlm.nih.gov/home/genomes/, accessed on 26 August 2024) was used to conduct Blastp analysis and gene annotation and then concluded the gene clusters of secondary metabolites in *A. manoharacharyi* NFCCI 5738.

### 2.10. Comparative Genomics and Phylogenetic Analysis

Orthologous proteins were identified using OrthoFinder version 2.5.5 [38] and the results were used to build a species tree in MEGA 11 using ML. For visualization and editing of the tree, iTOL: Interactive Tree of Life version 6.9 was used [39]. The taxa used for studying the orthologous proteins and construction of the phylogenetic tree are presented in Table 2. The average nucleotide identity (ANI) analysis was performed using the Pyani script and ANIb as an algorithm for the alignment [40]. The taxa used in the analysis of the ANI are presented in Table 2. Another ML phylogenetic tree was built using raxmlHPC v7.2.8 on the core genome SNPs identified in a pan-genome analysis performed using Panseq v3.2.1, with the run mode set to pan, the fragment size at 500 nucleotides, the percentage of identity cut off at 90%, and core genome threshold set at 2 to find out the sequences in common among all the taxa [41].

### 2.11. Characterization of Transcripts

The putative secreted proteins involved in pathogenesis were identified using SignalP version 5.0b with the cutoff set ≥ 0.5 [42]. TargetP version 2.0 was used to identify the signal peptide (SP), mitochondrial transit peptide (mTP), and potential cleavage sites (CSs) [43]. The prediction of transmembrane proteins was performed using TMHMM version 2.0 [44]; for the prediction of non-coding RNA, tRNAscan, RNAmmer, and rfam_scan were used.

### 2.12. Fermentation and Extraction of Metabolites of the Isolate for Metabolomic Study

*Alanomyces manoharacharyi* was initially grown on PDA for four days. Then, a single colony from PDA was inoculated in 1.2 L (400 mL × 3) potato dextrose broth in a 2 L Erlenmeyer flask (3 No.) at 180 rpm at 26 °C for a week. Later, the biomass was separated from the filtrate using Whatman No. 4 filter paper. The filtrate was extracted twice with an equal volume of ethyl acetate. The ethyl acetate extract was dried in a rotatory evaporator. The dried extract was dissolved in methanol. The biomass obtained was rinsed twice with distilled water and filtered. The biomass was dried at 45 °C and extracted overnight using methanol. Later, this biomass and methanolic mixture was subjected to sonication and filtered through Whatman No. 4 filter paper. This filtered methanolic extract was dried in a rotatory evaporator. The dried extract was dissolved in methanol. The extracts were diluted to a concentration of 1 mg/mL for further analysis.

### 2.13. Sample Preparation for Untargeted Metabolomics

In total, 100 μL of the sample was taken and 10 μL internal standard (ISTD) was added, followed by the addition of 400 μL Extraction Agent 1 (4 times the sample). The mixture was vortexed and mixed thoroughly. The incubation was carried out on ice for 20 min. After incubation, the sample was vortexed again and centrifuged at 14,000× *g* for 15 min at 4 °C. Without disturbing the pellet, 200 μL supernatant was carefully transferred to a fresh tube, followed by the addition of 800 μL Extraction Agent 1 and 200 μL Extraction Agent 2. The mixture was vortexed and mixed thoroughly. The supernatant was dried under a nitrogen beam and reconstituted in 200 μL RS Buffer. A 0.2-micron filter was wetted with 100 μL Extraction Agent 2 and the concentrated sample was filtered (Metabolomics Kit Catalog no. 912308). The filtered samples were collected into the autosampler vial. This method was targeted for both samples, i.e., filtrate and biomass extract.

### 2.14. UHPLC and MS Parameters

The metabolomic analysis was conducted using an Ultra-High-Performance Liquid Chromatography (UHPLC) system (Elute UHPLC, Bruker, Billerica, MA, USA). This system was equipped with a quaternary pump coupled with an Ion Trap mass spectrometer (Amazon Speed, Bruker) that utilized an Electrospray Ionization (ESI) interface. Chromatographic separation was achieved using an Acquity BEH C18 reversed-phase column (50 × 2.1 mm, 1.7 μm particle size) (Waters, Milford, MA, USA). The column was maintained at a temperature of 30 °C. The mobile phases consisted of an aqueous solution of 5 mM ammonium acetate and formic acid (FA), used in both positive and negative modes. The flow rate was kept constant at 0.3 mL/min. The gradient elution was optimized to start with 95% of Mobile Phase A, followed by a linear decrease to 25% of Mobile Phase B over 18 min. This was then reduced to 2% B over the next 5 min, held for 7 min, and then switched to 95% B. The column was equilibrated to the initial conditions over the next 5 min, resulting in a total analysis time of 35 min. The injection volume for the samples was set at 5 μL. The ESI-MS/MS analysis was performed in AUTO MSn mode, operating in positive and negative polarities. Two MS/MS transitions were acquired per analyte, with a dwell time between 0.017 and 0.130 s. The maximum accuracy time in the trap control section was 50 ms and the scan range was 100 to 2000 *m*/*z*. The nebulizer temperature was set at 29.0 psi, the dry gas flow rate was 10 L/min, and the dry temperature was 126.9 °C. The capillary voltage and end plate offset were set at 4500V and 500V, respectively. The ICC target in negative mode was 70,000 and in positive mode was 200,000.

### 2.15. Data Analysis Pipeline for Metabolomics

The pipeline began with the normalization of raw data on features, ensuring the preservation of data quality. Low-quality peaks were disqualified and the data were then cross-referenced against multiple databases, such as PubChem CID, CHEBI ID, HMDB ID, KEGG ID, ChemSpider ID, METLIN ID, BMRB ID, MetaCYC ID, Plant Metabolite Hub (Pmhub), YMDB ID, DRUGBANK, and LIPID MAPS. The entire mass of data generated was filtered to find biological features. The best-matched data generated were filtered to find biologically relevant features. The best-matched data were then tabulated for abundance.

## 3. Results

### 3.1. Phylogenetic Analysis

**Figure 1:**
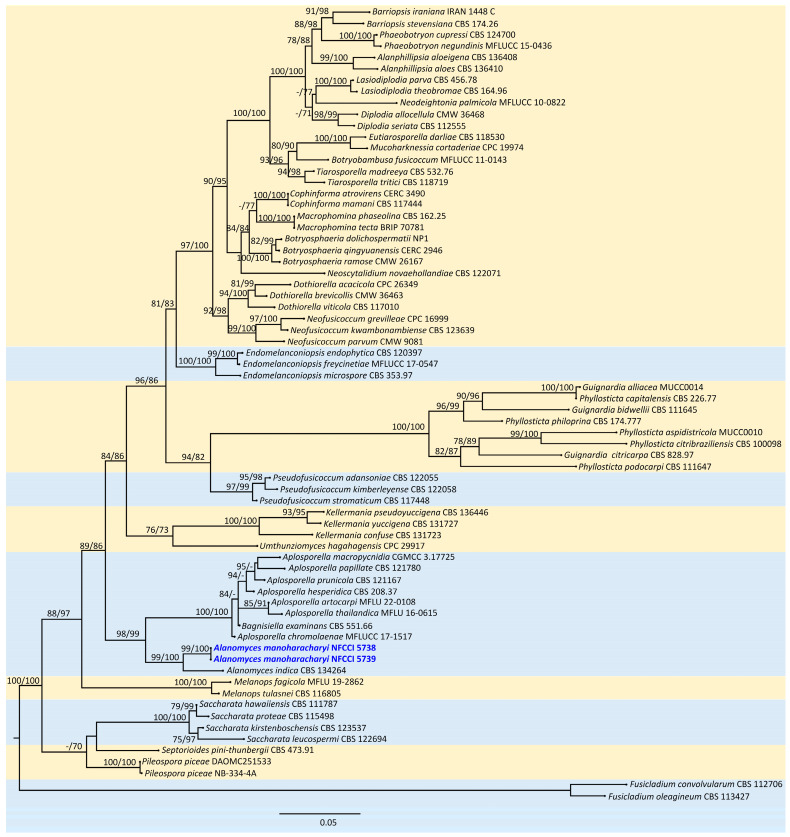
Molecular phylogenetic analysis of the new species *Alanomyces manoharacharyi* based on the ML method using combined ITS and LSU sequence data. The new species is shown in blue. Statistical support values of 70% or more are displayed next to each node and UFBS values and SH−aLRT are obtained from 1000 replicates using IQ−TREE and the TIM2e + I + G4 model.

The sequence alignments of ITS and LSU were used to confirm the identity of this isolate. The concatenated file had sequence data of 70 taxa (Table 1). The alignment contained 1644 columns, 480 parsimony-informative sites, 760 distinct patterns, 150 singleton sites, and 1014 constant sites. TIM2e + I + G4 was considered the best model and was selected based on the Bayesian Information Criterion (BIC). The phylogenetic tree was generated using the ML method based on the above-mentioned model. The log-likelihood of the consensus tree was −13128.93. Rate parameters were A–C: 1.27194, A–G: 2.38395, A–T: 1.27194, C–G: 1, C–T: 4.41005, and G–T: 1; base frequencies were A: 0.25, C: 0.25, G: 0.25, and T: 0.25; and the proportion of invariable sites was 0.489 and the gamma shape alpha parameter was 0.65 (Figure 1).

Combined phylogenetic analysis using ITS and LSU nested the *Alanomyces manoharacharyi* isolate in a distinct and unique clade in the family *Aplosporellaceae*. The clade was well supported with robust SH-like aLRT and ultrafast bootstrap (UFBoot) (Figure 1).

### 3.2. Taxonomy

**Figure 2:**
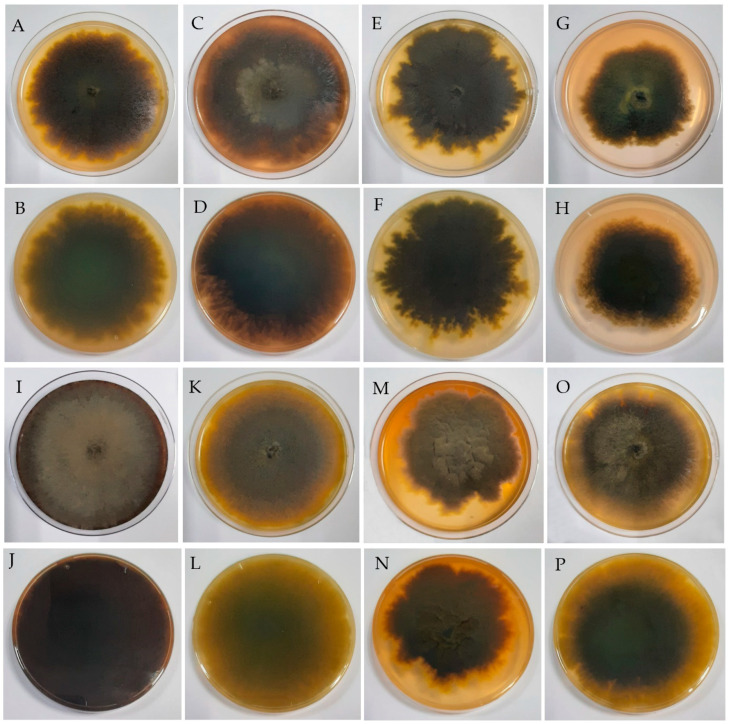
Colonies on various media after 10 days. (**A**,**B**) MEA; (**C**,**D**) V8 juice agar; (**E**,**F**) CMA; (**G**,**H**) RBA; (**I**,**J**) CDA; (**K**,**L**) PCA; (**M**,**N**) SDA; (**O**,**P**) PDA; (**A**,**C**,**E**,**G**,**I**,**K**,**M**,**O**) front view; (**B**,**D**,**F**,**H**,**J**,**L**,**N**,**P**) reverse view.

**Figure 3:**
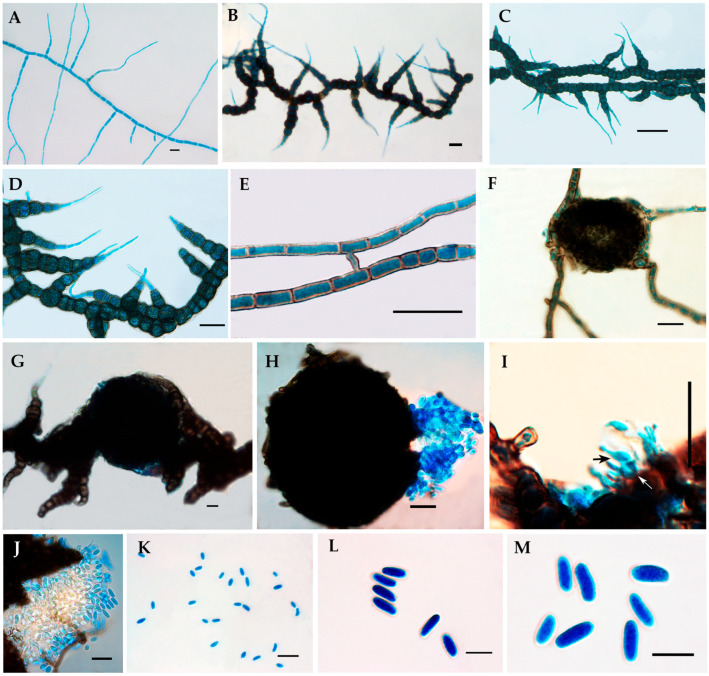
*Alanomyces manoharacharyi* NFCCI 5738; (**A**–**D**) Hyphae; (**E**) Hyphae showing anastomosis; (**F**,**G**) Conidiomata; (**H**) Ruptured conidiomata; (**I**) Ruptured conidiomata showing numerous dense conidiophores; the black arrow shows ampulliform conidiogenous cells; the white arrow shows short, stumpy conidiophores; (**J**) Ruptured conidiomata with numerous conidia; (**K**–**M**) Conidia. Bar = 20 µm (**A**–**K**), 10 µm (**L**,**M**).

*Alanomyces manoharacharyi* S. Rana and S.K. Singh, sp. nov. Figure 2 and Figure 3.

MycoBank Number: MB 854034.

Holotype: AMH 10702.

Etymology: Named in honor of Prof. Chakravarthula Manoharachary, an eminent mycologist from India.

Host/distribution: Endophyte bark of *Azadirachta indica* collected from Mulshi, Pune, Maharashtra, India.

Original description: Hyphae: branched, septate, pigmented, constricted near septa, wall thickened and darkened, rough walled, lateral hyphae narrowing towards the apex, dark olivaceous brown, hyaline towards apex, 1.2–33.5 μm ($\bar{x}$ = 13.2 μm, *n* = 30). Conidiomata: pycnidial, abundantly produced, globose to subglobose to irregular, dark brown to blackish brown, outer layer composed of dark brown *textra angularis*, nonsetose, 40.5–246.4 × 35.4–218.4 μm ($\bar{x}$ = 111.4 × 98.7 μm, *n* = 30). Conidiophores: short, stumpy, reduced in size, and hyaline. Conidiogenous cells: terminal, integrated, phialidic, ampuliform, smooth-walled, aseptate, hyaline, 11.6–20 μm ($\bar{x}$ = 16.3 μm, *n* = 30). Conidia: cylindrical, apex broadly fusoid, base sub-rounded to rounded, smooth-walled, aseptate, hyaline, 6.0–14.6 × 3.5–6 μm ($\bar{x}$ = 11.1 × 4.8 μm, *n* = 30).

Culture characteristics: Colonies on PDA reaching 80 mm diam. after 10 days, at 25 °C: irregular, cottony, slightly raised, margins undulate; front olive brown (4D8), reverse smoke brown (4F2) to olive brown (4D8). Colonies on SDA reaching 70 mm diam. after 10 days, at 25 °C: velvety, sulcate, flat, margins irregular to undulate; front brownish grey (11F2) to (11D2), reverse sepia brown (4F3) to butter yellow (4A5). Colonies on PCA reaching 85 mm diam. after 10 days, at 25 °C: circular, flat, margins filiform to undulate; front sepia (brown) (4F3) to khaki (4D5), reverse goose turd (3F4) to olive (3D5). Colonies on CDA reaching 85 mm diam. after 10 days, at 25 °C: flat, irregular, velvety, margins filiform to undulate; front bluish grey (20D2), reverse purplish grey (13F3). Colonies on RBA reaching 65 mm diam. after 10 days, at 25 °C: flat, irregular, velvety, margins filiform to undulate; front olive brown (4E4), reverse smoke brown (4F2) to olive brown (4F8). Colonies on CMA reaching 78 mm diam. after 10 days, at 25 °C: flat, irregular, cottony, margins irregular to undulate; front sepia brown (4F3) to olive brown (4E6), reverse smoke brown (4F2). Colonies on V8 juice agar reaching 85 mm diam. after 10 days, at 25 °C: flat, irregular, cottony, margins undulate; front Café au lait (6D2) to negro (6F3), reverse grey (6F1) to teak brown (6F5). Colonies on MEA reaching 80 mm diam. after 10 days, at 25 °C: flat, irregular, cottony, margins undulate; front sepia (brown) (4F3) to yellow (3A6), reverse goose turd (3F3) to pastel yellow (3A4) (Figure 2).

Sexual morph: Not observed.

Known distribution: Mulshi, Pune, Maharashtra, India.

Material examined: INDIA, Maharashtra, Pune, Mulshi, from the bark of Azadirachta indica, S.K. Singh, 11 February 2024, AMH 10702 (holotype), deposited in Ajrekar Mycological Herbarium (AMH) of India, ex-type culture is deposited in the National Fungal Culture Collection of India (NFCCI 5738).

GenBank numbers: PP669818 (ITS) and PP669820 (LSU).

Other specimens examined: India, Maharashtra, Pune, Mulshi, *Azadirachta indica*, S.K. Singh, 11 February 2024, NFCCI 5739; GenBank numbers: PP669819 (ITS), PP669821 (LSU).

Notes: The family *Aplosporellaceae* currently possesses two genera; one is *Aplosporella* Speg. [9] and the second one is *Alanomyces* Sharma [10]. The literature review indicates that the genus *Alanomyces* was recently established by Sharma et al. [10] with the type species *Alanomyces indica* Sharma. The comparison of the morphotaxonomic features of our new collection, *Alanomyces manoharacharyi*, reveals that it is morphologically different from the type species *A*. *indica*. The pycnidia are significantly smaller without setae in the present collection 40.5–246.4 × 35.4–218.4 μm ($\bar{x}$ = 111.4 × 98.7 μm, n = 30) while larger and setose in the type species *A*. *indica* 100–200 × 10–12 μm. The conidia/pycniospores were prominently found in the ruptured pycnidia/conidiomata. The conidiogenous cells in *A*. *manoharacharyi* were prominently found in the juvenile conidia/pycniospores produced in Figure 3I.

In addition to the morphological characteristics, *Alanomyces manoharacharyi* and *A*. *indica* differ in their habitat too. The present collection was isolated as an endophyte from the bark of *Azadirachta indica* while *Alanomyces indica* was isolated from soil attached to the fruiting body of an unidentified macrofungus.

Based on the MegaBLAST algorithm search on NCBI for *Alanomyces manoharacharyi* NFCCI 5738, the closest hit using the ITS gene sequence was found to be *Bagnisiella examinans* CBS 551.66 showing 93.15% (884 out of 949 bp) identity and having twenty-one gaps (2.21%), with *Aplosporella prunicola* CBS 121167 showing 92.44% (856 out of 926 bp) identity and having twenty-seven gaps (2.92%), and with *Alanomyces indica* CBS 134264 showing 94.73% (557 out of 588 bp) identity and having nine gaps (1.53%).

Based on the distinguished morphological features, and phylogenetic analysis, the present collection, *Alanomyces manoharacharyi*, is treated here as a novel species of *Alanomyces* (second in the genus).

### 3.3. Identification of the Isolate by MALDI-TOF Mass Spectrometry

**Figure 4:**
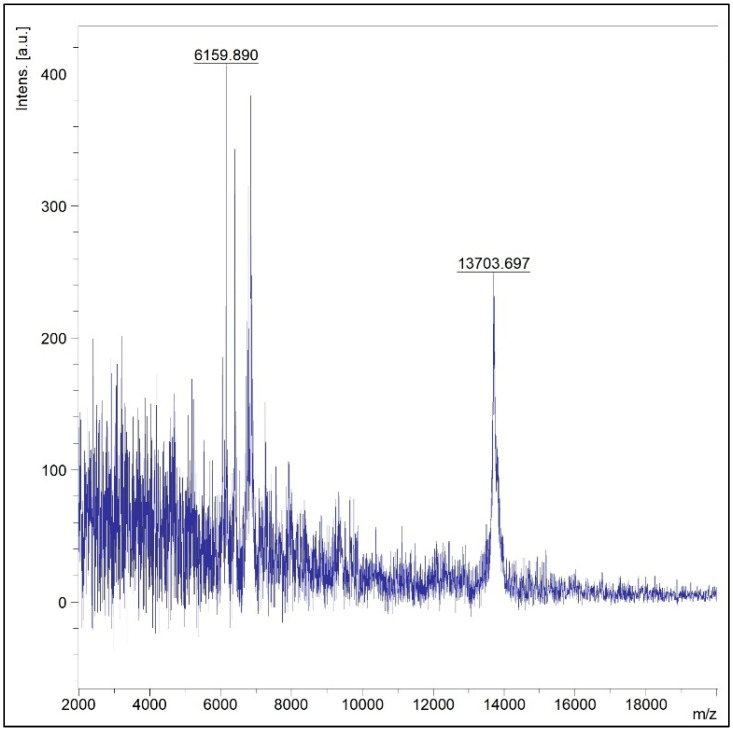
MALDI-TOF MS spectra of *Alanomyces manoharacharyi* NFCCI 5738 indicating the protein profile (2–20 KD).

MALDI-TOF MS spectra of the protein profile (2–20 KD) of *Alanomyces manoharacharyi* NFCCI 5738 were studied using MALDI-TOF Mass Spectrometry (Figure 4 and Table 3). Interestingly, and as expected, the results displayed “No organism identification possible” with a score value of 1.17 as this isolate is novel and did not match with the existing species listed available in the database. MALDI-TOF MS spectra also indicate the novelty of the isolate.

### 3.4. Genome Sequencing and Assembly of Alanomyces manoharacharyi NFCCI 5738

The sample passed the QC threshold (Q30 > 85%). The total number of raw reads generated was 150169230, GC% was 46, and %Q30 was 94.3. The number of reads that passed the quality check was 148,991,456. Based on the assembly statistics from the assemblers MaSurCa 4.0.5, Megahit v1.2.9, and Spades v3.15.4, it was determined that the Spades assembler yielded the best results (Table 4). Spades assembly was used further for downstream analysis.

The complete number of BUSCOs (C) was 1677 (98.3%), complete and single-copy BUSCOs (S) was 1674 (98.1%), complete and duplicated BUSCOs (D) was 9 (0.2%), fragmented BUSCOs was (F) 7 (0.4%), missing BUSCOs (M) was 22 (1.3%), and total BUSCO groups searched was 1706.

**Figure 5:**
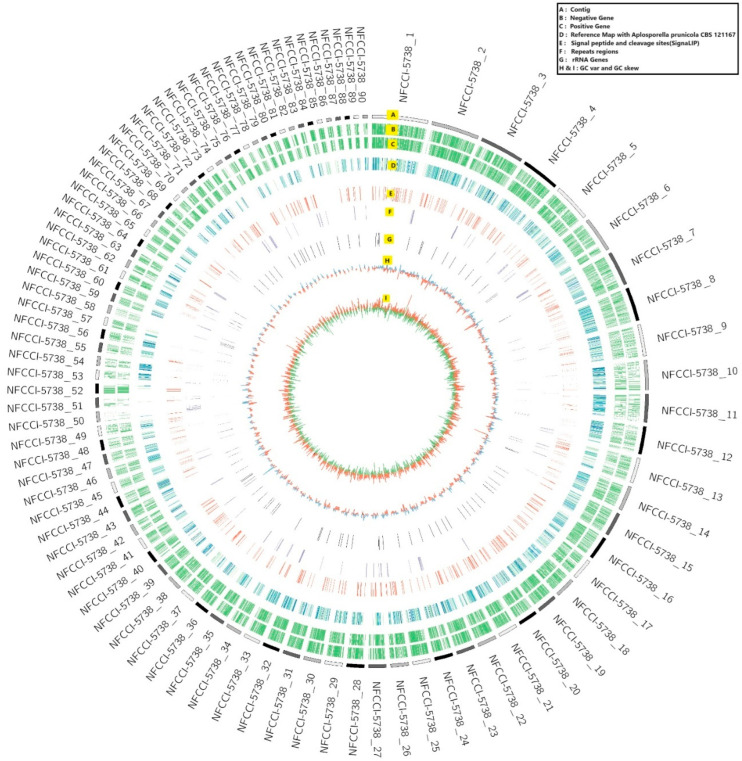
Genome diagram of *Alanomyces manoharacharyi* NFCCI 5738; A: Contig; B: Negative Gene; C: Positive Gene; D: Reference Map with *Aplosporella punicola* CBS 121167; E: Signal Peptide with cleavage sites (Signal LIP); F: Repeat regions; G: rRNA Genes; H: GC variation and I: GC skew.

The genome sequence of *Alanomyces manoharacharyi* NFCCI 5738 was assembled and deposited in the NCBI GenBank database (BioProject PRJNA1114393; BioSample SAMN41484321). To represent the genome of *A. manoharacharyi* NFCCI 5738, the contigs were sorted from largest to smallest and the top 90 contigs were represented using CIRCOS as a genome diagram. The genome diagram of *A. manoharacharyi* NFCCI 5738 shows that there are nine circles in the circle diagram (Figure 5), which are as follows from inside to outside: the first circle in orange and green (I) shows the GC skew, the second circle in red and blue (H) represents GC variation, the third circle in black (G) represents rRNA genes, the fourth circle in purple (F) represents repeat regions, the fifth circle in red (E) represents the signal peptide and cleavage site (Signal LIP), the sixth circle in blue and green (D) represents the reference map with *Aplosporella prunicola* CBS 121167, the seventh circle in green (C) indicates that CDS is in a positive chain, and the eighth circle in green (B) indicates that CDS is in a negative chain. The outer rim shows the contigs.

The genome length was 35,009,973 bp. The total number of contigs generated was 264. The total contigs length was 35,550,828 bp. Contig maximum length was 1,303,379 bp. GC content was found to be 50.01%. The N50 value was found to be 408,258 bp. The Augustus prediction method was used to predict the encoding genes; in total, 7870 protein-coding genes were predicted. The length was 3,902,274 bp. The gene’s average length was 495.8416773 bp. The gene length/genome was 10.976605. GC content in the gene region was 54.06%. In addition to this, the gene prediction was also conducted using GeMoMa in which the reference genome used for the prediction of genes was *Aplosporella prunicola* CBS 121,167. As mentioned earlier, the number of genes predicted using Augustus was 7870; similarly, the number of genes predicted using GeMoMa was 8101, the number of genes only predicted by Augustus was 6201, the number of genes predicted by GeMoMa was 6432, and the number of genes which were found to be common and predicted by both Augustus and GeMoMa was 1669 (Supplementary Materials sheet attached for further details).

### 3.5. Genome Sequence Annotation of Alanomyces manoharacharyi NFCCI 5738

Simultaneously, the three prediction methods, Proteinmask, and RepeatMasker were used to predict repeated sequences. Proteinmask predicted that the number of repeating sequences was 1832, occupying 4.65% of the whole genome, and RepeatMasker predicted that the number of repeating sequences was 13437, occupying 3.25% of the entire genome. For non-coding RNA, we predicted three-hundred and fourty-four secondary structures of RNA and tRNA by tRNAscan and three rRNA were predicted by RNAmmer. At the same time, 104 snRNA were predicted with the Rfam database by rfam_scan.

To predict the protein sequences, 7870 non-redundant genes of *A. manoharacharyi* NFCCI 5738 were subjected to a similarity search based on various public databases. Many genes were mapped using the Clusters of Orthologous Groups (COGs) database (3504 genes/44.52%), the Kyoto Encyclopedia of Genes and Genomes (KEGG) (2836 genes/36.04%), Gene Ontology (GO) (4064 genes/51.64%), Swiss-Prot (4094 genes/52.02%), NCBI non-redundant nucleotide sequences (NTs) (1983 genes/25.20%), and NCBI non-redundant protein sequences (NRs) (6461 genes/82.10%) and, overall, (6885 genes/87.48%) were annotated.

**Figure 6:**
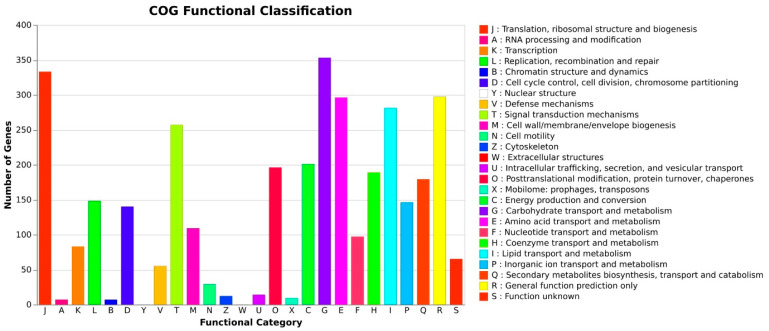
Functional annotation of *Alanomyces manoharacharyi* NFCCI 5738 genes encoding for proteins using the Clusters of Orthologous Genes (COGs) database.

As per the COG database, “Carbohydrate transport and metabolism” was related to many genes (353), followed by “Translation, ribosomal structure and biogenesis (333)”, “General function prediction only (297)”, “Amino acid transport and metabolism (296)”, “Lipid transport and metabolism (281)”, and “Signal transduction mechanisms (258)” (Figure 6) [45]. These results depict that *A. manoharacharyi* NFCCI 5738 possesses a varied and enriched array of functions for carbohydrates, amino acid metabolism, and lipid transport and metabolism that may favor better energy conversion efficiency.

**Figure 7:**
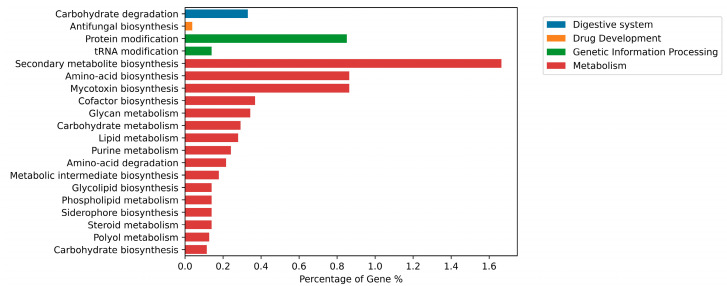
Functional annotation of *Alanomyces manoharacharyi* NFCCI 5738 genes encoding for proteins using Kyoto Encyclopedia of Genes and Genomes (KEGG) analysis.

The findings from the KEGG functional classification suggest that the predicted proteins fell under various categories, such as digestive system (26) [carbohydrate degradation (26)]; drug development (3) [antifungal biosynthesis (3)]; genetic information processing (80) [protein modification (67), tRNA modification (11), bacterial outer membrane biogenesis (1), cell wall biogenesis (1)]; and metabolism (635) [secondary metabolite biosynthesis (131), amino-acid biosynthesis (68), mycotoxin biosynthesis (68), cofactor biosynthesis (29), glycan metabolism (27), carbohydrate metabolism (23), lipid metabolism (22), purine metabolism (19), amino-acid degradation (17), metabolic intermediate biosynthesis (14), glycolipid biosynthesis (11), phospholipid metabolism (11), siderophore biosynthesis (11), steroid metabolism (11), polyol metabolism (10), carbohydrate biosynthesis (9), pigment biosynthesis (9), sulfur metabolism (9), carbohydrate acid metabolism (8), nucleotide-sugar biosynthesis (8), pyrimidine metabolism (8), polyketide biosynthesis (7), xenobiotic degradation (7), alkaloid biosynthesis (6), amine and polyamine biosynthesis (6), aromatic compound metabolism (6), nitrogen metabolism (6), one-carbon metabolism (6), porphyrin-containing compound metabolism (6), energy metabolism (4), glycan degradation (4), isoprenoid biosynthesis (4), protein biosynthesis (4), sesquiterpene biosynthesis (4), steroid biosynthesis (4), glycan biosynthesis (3), glycerolipid metabolism (3), hormone biosynthesis (3), organic acid metabolism (3), alcohol metabolism (2), amino-acid metabolism (2), antibiotic biosynthesis (2), carotenoid biosynthesis (2), cofactor degradation (2), cofactor metabolism (2), organosulfur degradation (2), phytoalexin biosynthesis (2), alkaloid degradation (1), amine and polyamine degradation (1), amino-sugar metabolism (1), flavonoid metabolism (1), ketone metabolism (1), membrane lipid metabolism (1), phytotoxin biosynthesis (1), plant hormone metabolism (1), secondary metabolite metabolism (1), sphingolipid metabolism (1)] (Figure 7) [46]. The results indicate that a varied and enriched array of metabolic functions is present that will probably provide higher secondary metabolism efficacy.

**Figure 8:**
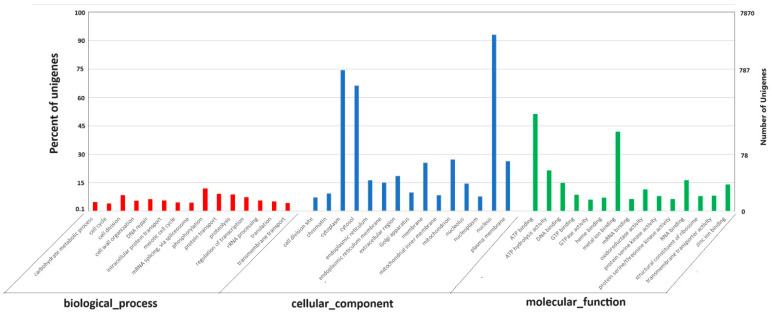
Functional annotation of *Alanomyces manoharacharyi* NFCCI 5738 predicted genes encoding for proteins using Gene Ontology (GO) analysis; Red bars represent biological processes, blue bars represent cellular component and green represent molecular function.

GO annotation depicts varied genes possessed by *A. manoharacharyi* NFCCI 5738, which may be involved in biological processes, molecular functions, and cellular components (Figure 8) [47]. In total, 1003 (12.74%) genes were involved in biological processes, which included genes involved in the carbohydrate metabolic process (50), cell cycle (42), cell division (89), cell wall organization (57), DNA repair (66), intracellular protein transport (59), meiotic cell cycle (48), mRNA splicing via spliceosome (46), phosphorylation (124), protein transport (95), proteolysis (92), regulation of transcription (77), rRNA processing (59), translation (54), and transmembrane transport (45). A total of 4397 (55.87%) genes were involved in cellular component function, which included genes involved in the cell division site (75), chromatin (97), cytoplasm (780), cytosol (694), endoplasmic reticulum (170), endoplasmic reticulum membrane (157), extracellular region (194), Golgi apparatus (103), membrane (267), mitochondrial inner membrane (89), mitochondrion (285), nucleolus (152), nucleoplasm (81), nucleus (976), and plasma membrane (277). A total of 2403 (30.53%) genes were involved in molecular function, which included genes involved in ATP binding (537), ATP hydrolysis activity (226), DNA binding (156), GTP binding (90), GTPase activity (63), heme binding (73), metal ion binding (440), mRNA binding (67), oxidoreductase activity (119), protein serine kinase activity (82), protein serine/threonine kinase activity (66), RNA binding (171), structural constituent of ribosome (82), transmembrane transporter activity (85), and zinc ion binding (146).

### 3.6. Genome Sequence Annotation of Alanomyces manoharacharyi NFCCI 5738 for Carbohydrate Genes

**Figure 9:**
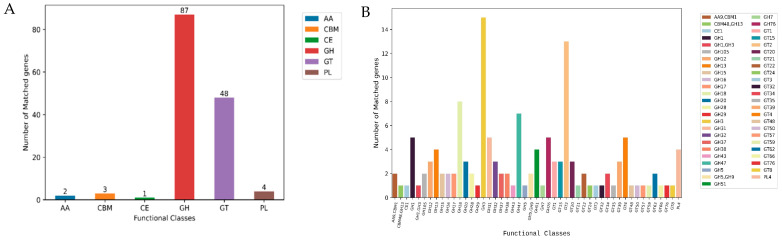
Carbohydrate-active enzyme (CAZyme) functional classification and corresponding genes present in the *Alanomyces manoharacharyi* NFCCI 5738 genome. (**A**): Carbohydrate-active enzyme functional classes; (**B**): Carbohydrate-active enzyme functional subclasses.

Carbohydrate-active enzymes (CAZymes) represent a diverse group of enzymes responsible for both breaking down and building up glycoconjugates, as well as various forms of glycans, such as oligosaccharides and polysaccharides [48]. These enzymes are pivotal in fungal metabolism, facilitating carbohydrates’ degradation, modification, and biosynthesis [49]. The CAZy database is a specialized resource for carbohydrate enzymes, encompassing their ability to alter, create, and dismantle glycosidic bonds [49]. The analysis showed 145 genes encoded for CAZymes dispersed in the *A. manoharacharyi* NFCCI 5738 genome. These include two auxiliary activities (AAs), three carbohydrate-binding modules (CBMs), one group of carbohydrate esterases (CEs), eighty-seven glycoside hydrolases (GHs), fourty-eight glycosyltransferases (GTs), and four polysaccharide lyases (PLs; Figure 9A). Details of the families are shown in Figure 9B. These findings show that *A. manoharacharyi* NFCCI 5738 can show a remarkable ability to make and break complex carbohydrates and is possibly a candidate for industrial applicability.

### 3.7. Genome Sequence Annotation of Alanomyces manoharacharyi NFCCI 5738 for Pathogen–Host Interactions

**Figure 10:**
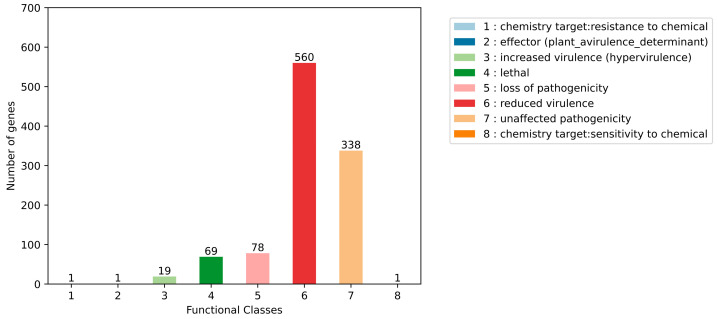
Distribution map of mutation types in the pathogen PHI phenotype of *Alanomyces manoharacharyi* NFCCI 5738.

The Pathogen–Host Interactions Database (PHI-base) is an extensively curated database constructed by experts, relying on experimental data. It contains genes associated with virulence, effector molecules, and pathogenicity factors derived from various pathogens, including fungi, bacteria, and oomycetes. These pathogens infect multiple hosts, including plants, animals, insects, and fungi [50]. The amino acid sequences of *A. manoharacharyi* NFCCI 5738 were compared with the PHI-base. As shown in Figure 10, *A. manoharacharyi* NFCCI 5738 possesses abundant PHI-base genes, including reduced virulence (560), unaffected pathogenicity (338), loss of pathogenicity (78), lethal (69), increased virulence (hypervirulence; 19), sensitivity to chemicals (1), effector (plant avirulence determinant; 1), and resistance to chemicals (1) [51]. Reduced virulence, unaffected pathogenicity, and loss of pathogenicity were the significant annotation genes indicating that *Alanomyces manoharacharyi* NFCCI 5738 is not a pathogenic strain as expected as it was isolated as an endophyte and can be targeted without hesitation for industrial application.

### 3.8. AntiSMASH Analysis of Alanomyces manoharacharyi NFCCI 5738

**Figure 11:**
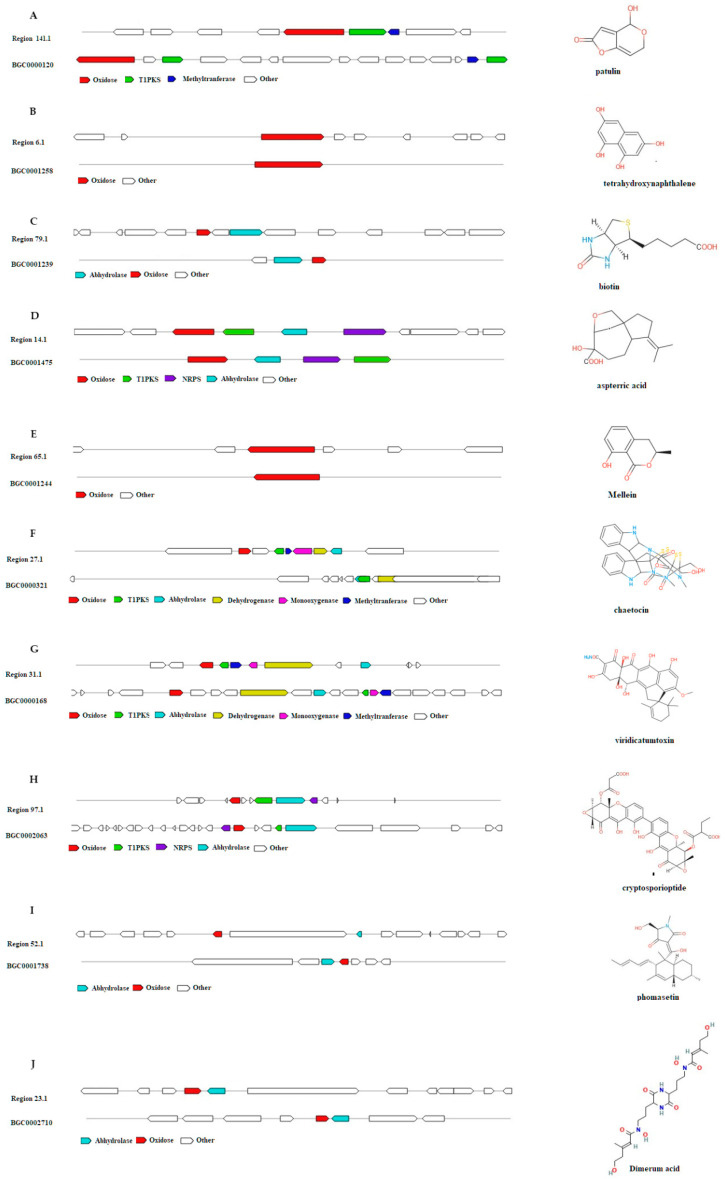
Comparison of biosynthetic gene cluster components in *Alanomyces manoharacharyi* NFCCI 5738 with known biosynthetic gene clusters for the biosynthesis of (**A**) Patulin; (**B**) Tetrahydroxynaphthalene; (**C**) Biotin; (**D**) Aspterric acid; (**E**) Mellein; (**F**) Chaetocin; (**G**) Viridicatumtoxin; (**H**) Cryptosporioptide; (**I**) Phomasetin; and (**J**) Dimerum acid.

AntiSMASH analysis indicated that *A. manoharacharyi* isolate NFCCI 5738 contains twenty-six secondary metabolite biosynthetic gene clusters (BGCs), including three terpenes, six Type I PKSs (polyketide synthases; T1PKS), one beta-lactone-containing protease inhibitor (beta lactone), seven nonribosomal peptide synthetases (NRPSs), six NRPS-like fragments (NRPS-like), one fungal RiPP-like, one hybrid NRPS + indole, and one hybrid NRPS + T1PKS (Table 5 and Figure 11). AntiSMASH results revealed the potential of this isolate to produce exciting compounds, such as 1,3,6,8-tetrahydroxynaphthalene, aspterric acid, metachelin C/metachelin A/metachelin A-CE/metachelin B/dimerumic acid 11-mannoside/dimerumic acid, chaetocin, viridicatumtoxin/previridicatumtoxin/5-hydroxyanthrotainin/8-O-desmethylanthrotainin, phomasetin, (-)-Mellein, cryptosporioptide B/cryptosporioptide A/cryptosporioptide C, 11-mannoside/dimerumic acid, biotin, and patulin.

Only eleven BCGs showed homologies with known clusters, of which three BCGs showed 100% similarity with known clusters, i.e., 1,3,6,8-tetrahydroxynaphthalene, aspterric acid, and (-)-Mellein. Almost 58% of BGCs did not match with any known gene clusters, indicating that there are many unknown products to be explored and that *A. manoharacharyi* NFCCI 5738 has the potential to biosynthesize more novel compounds. Genes within the region 6.1 (BGC 6.1) (7560 nucleotides) displayed 100% similarity with the 1,3,6,8-tetrahydroxynaphthalene biosynthetic gene cluster from the *Glarea lozoyensis* (Figure 11) BGC (MIBiG: BGC0001258; NCBI GenBank: AF549411.1) [52]. Genes within the region 14.1 (BGC 14.1) (21,193 nucleotides) displayed 100% similarity with the aspterric acid biosynthetic gene cluster from the *Aspergillus terreus* NIH2624 (Figure 11) BGC (MIBiG: BGC0001475; NCBI GenBank: NT_165929.1) [53]. Genes within the region 65.1 (BGC 65.1) (45,412 nucleotides) displayed 100% similarity with the aspterric acid biosynthetic gene cluster from the (-)-Mellein biosynthetic gene cluster from the *Parastagonospora nodorum* (Figure 11) BGC (MIBiG: BGC0001244; NCBI GenBank: KM365454.1) [54].

Many of these metabolites have been reported earlier to be capable of possessing various bioactivities. Additionally, (-)-Mellein has been proven to be produced from termites and reported to display an inhibitory effect on the growth of entomopathogenic fungi (*Metarhizium anisopliae* and *Beauveria bassiana*) [55]. Additionally, 1,3,6,8-tetrahydroxynaphthalene has been reported to be a melanin precursor with many uses; it can protect against ionizing radiation, including ultraviolet, X-ray, gamma-ray, and particulate radiation. These melanins can be used to improve human health, environments, and industries [56]. In addition to this, aspterric acid and 6-hydroxymellein have been reported to inhibit pollen development in *Arabidopsis thaliana* [57]. Biotin (vitamin H or B7) has become the new trend for consumers wishing to have longer, healthier hair and nails [58]. Numerous studies have demonstrated a wide range of antitumor activities of chaetocin in vitro and in vivo. It has also been isolated and reported in *Chaetomium* species [59]. Cryptosporioptide is an antibiotic that has been reported to exhibit both lipoxygenase inhibitory and anti-*Bacillus megaterium* activities; it has been reported to be produced from the endophytic fungus *Cryptosporiopsis* sp. [60]. In the 1960s, patulin was used for treating common colds and nose infections because of its antiviral, antiprotozoal, and antibacterial properties; it has been mainly reported to be produced by fungal genera, like *Penicillium*, *Aspergillus*, and *Byssochlamys* [61]. Phomasetin is known to inhibit HIV-1 integrase and has been reported to be produced from *Phoma* sp.

### 3.9. Comparative Phylogenetics and Genomics

#### 3.9.1. Average Nucleotide Identity

**Figure 12:**
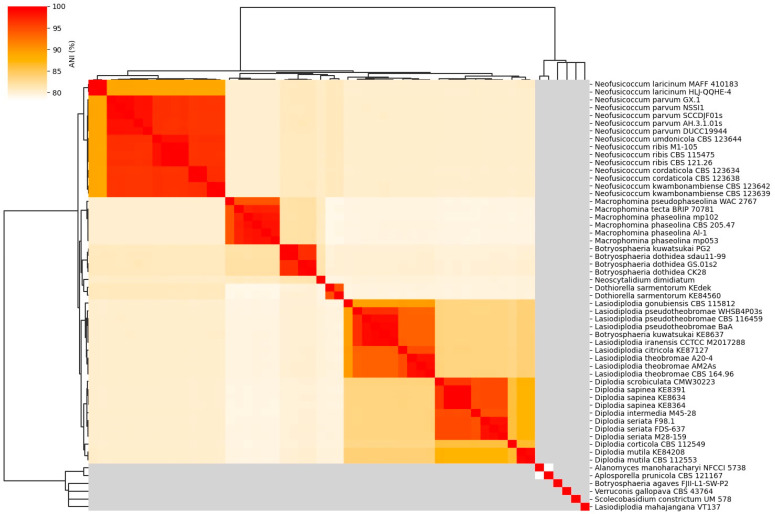
Heatmap of ANIb percentage identity between the allied genera strains compared with the *Alanomyces manoharacharyi* NFCCI 5738. ANIb analysis was carried out for all 55 genomes calculated based on genome sequences.

Average nucleotide identity (ANI) analysis performed on the *A. manoharacharyi* NFCCI 5738 genome provided an overall idea of the sequence identity between the allied genera in comparison with *A*. *manoharacharyi* strain NFCCI 5738, as depicted in the heatmap in Figure 12. *Alanomyces manoharacharyi* NFCCI 5738 was grouped with *Aplosporella prunicola* CBS 121167, thus confirming that it falls under the family *Aplosporellaceae*, order *Botryosphaeriales*. ANIb analysis included 55 genomes of various species from allied genera of *Alanomyces*.

#### 3.9.2. Phylogeny Based on Orthologous Proteins

**Figure 13:**
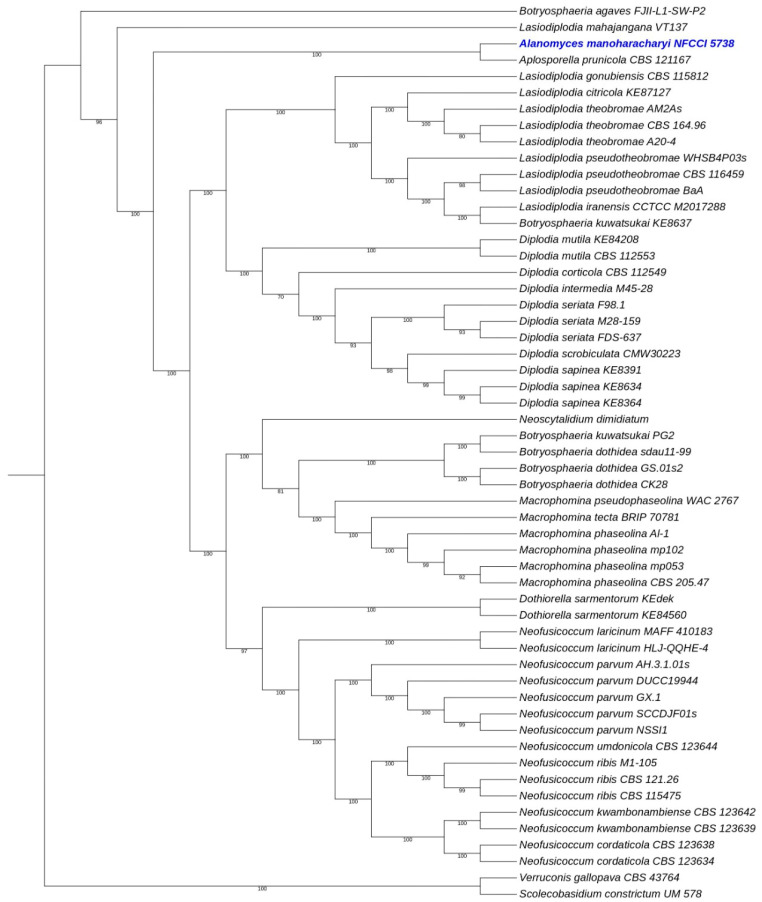
Phylogenetic analysis of 55 taxa of *Alanomyces manoharacharyi* NFCCI 5738 and allied taxa based on the orthologous proteins identified using OrthoFinder. The new species is shown in blue. Only the bootstrap values higher than 70 are shown.

*Alanomyces manoharacharyi* NFCCI 5738 was grouped with *Aplosporella prunicola* CBS 121167 in the phylogenetic tree, which was constructed based on the orthologous proteins of allied taxa of *Alanomyces* and figured out using OrthoFinder v2.5.5. This confirms the strain NFCCI 5738 clustering in the *Aplosporellaceae* family (Figure 13).

#### 3.9.3. Phylogeny Based on Single Nucleotide Polymorphisms (SNPs)

**Figure 14:**
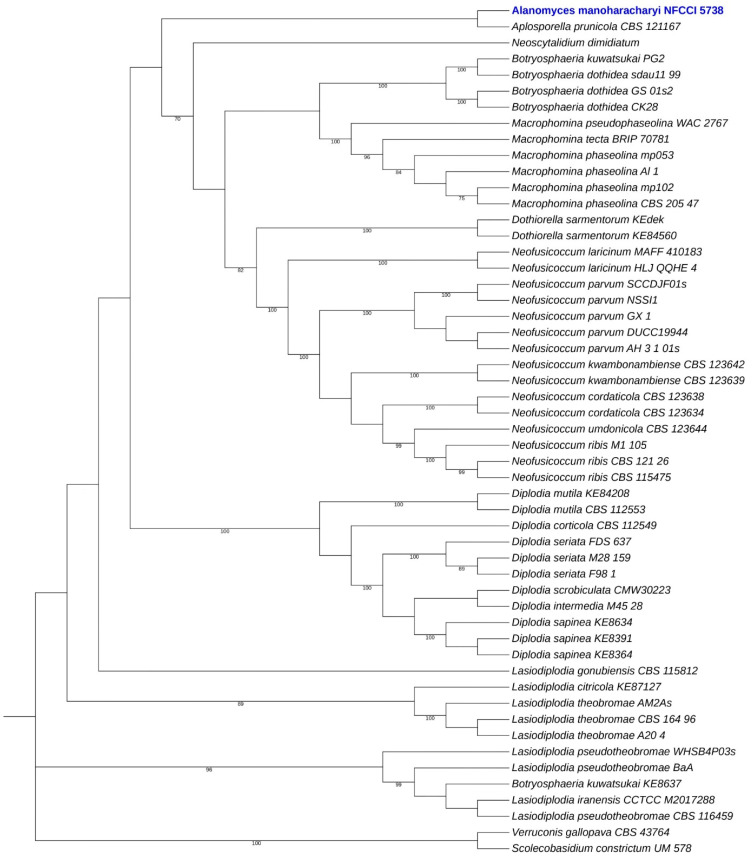
The maximum phylogenetic tree is based on the 130874 core genome SNPs identified using Panseq. The number of bootstraps is indicated as well. Only the bootstrap values higher than 70 are shown. The new species is shown in blue.

To further disentangle the phylogenetic relationship among the *A. manoharacharyi* NFCCI 5738 and allied taxa strains, a MLtree was built on 130874 SNPs found in the core genome alignment derived from Panseq. As illustrated in Figure 14, the clustering of *A. manoharacharyi* NFCCI 5738 with *Aplosporella prunicola* CBS 121167 was confirmed, thus confirming that *A. manoharacharyi* falls under the *Aplosporellaceae* family.

### 3.10. Characterization of Transcripts

**Figure 15:**
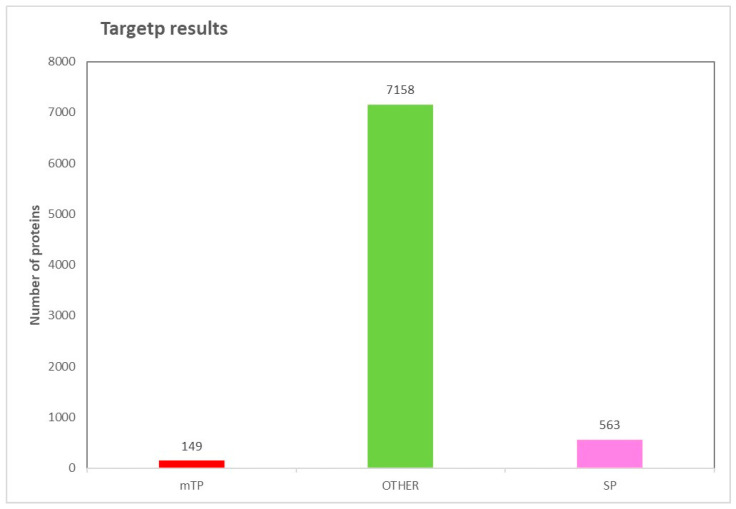
Results of TargetP analysis. Cumulative count of predicted proteins containing a signal peptide (SP), mitochondrial translocation signal (mTP), and no-targeting peptides (other).

One-thousand and sixty-six analogous genes were identified from the PHI-base. Among these, 449 genes were detected as putative secreted proteins using SignalP. TargetP helped to predict the presence of N-terminal presequences. It was found that there were 149 mitochondrial transit peptides (mTPs), 563 signal peptides (SPs), and 7158 no-targeting peptides (Other) (Figure 15).

### 3.11. Metabolites from Alanomyces manoharacharyi

**Figure 16:**
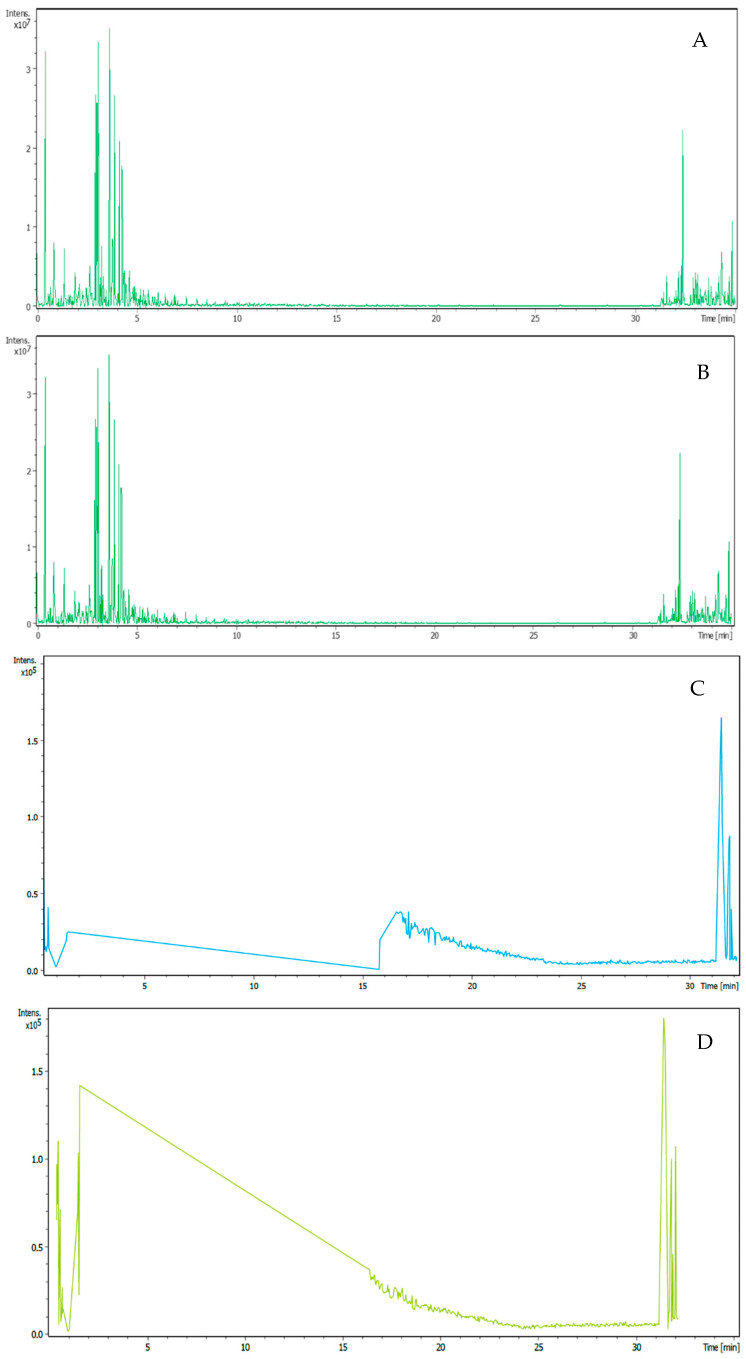
LC–MS analysis of extracts from *Alanomyces manoharacharyi* NFCCI 5738 for the identification of constituents. (**A**) Methanolic extract, Positive ion mode; (**B**) Ethyl acetate extract, Positive ion mode; (**C**) Methanolic extract, Negative ion mode; (**D**) Ethyl acetate extract, Negative ion mode.

**Figure 17:**
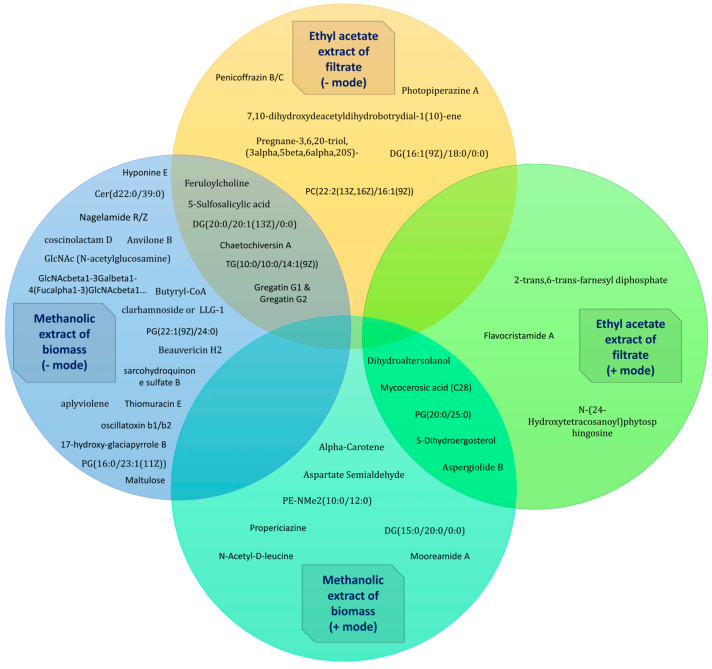
Metabolites identified from the methanolic extract of biomass and the ethyl acetate extract of the filtrate *Alanomyces manoharacharyi* NFCCI 5738 using LC–MS in positive and negative ion mode.

Metabolite profiling of the methanolic extract of the dried biomass and the ethyl acetate extract of the filtrate revealed a variety of compounds (Figure 16 and Figure 17; Table 6). Various interesting compounds, like Aspartate semialdehyde, N-Acetyl-D-leucine, PG(20:0/25:0), Dihydroaltersolanol and Dihydroaltersolanol C, Propericiazine (oxide), 2-trans,6-trans-farnesyl diphosphate, Mooreamide A, 5-Dihydroergosterol, Mycocerosic acid (C28), Aspergiolide B, Alpha-Carotene, PE-NMe2(10:0/12:0), DG(15:0/20:0/0:0), Flavocristamide A, N-(24-Hydroxytetracosanoyl)phytosphingosine, 5-Sulfosalicylic acid, Penicoffrazin B/C, Feruloylcholine, 7,10-dihydroxydeacetyldihydrobotrydial-1(10)-ene, Gregatin G1 and Gregatin G2, coscinolactam D, Photopiperazine A, 17-hydroxy-glaciapyrrole B, clarhamnoside or LLG-1, GlcNAcbeta1-3Galbeta1-4(Fucalpha1-3)GlcNAcbeta1…, Pregnane-3,6,20-triol, (3alpha,5beta,6alpha,20S), Chaetochiversin A, aplyviolene, Thiomuracin E, Cer(d22:0/39:0), GlcNAc (N-acetylglucosamine), CL(1′-[16:0/18:0],3′-[18:2(9Z,12Z)/20:4(5Z,8Z,1…, Anvilone B, Oscillatoxin b1/b2, DG(16:1(9Z)/18:0/0:0), TG(10:0/10:0/14:1(9Z)), DG(20:0/20:1(13Z)/0:0), Maltulose, Nagelamide R/Z, and Sarcohydroquinone sulfate B, were detected to be produced by *A. manoharacharyi*. Analysis of the crude methanolic extract of the dried biomass and the ethyl acetate extract of the filtrate from *A. manoharacharyi* showed moderate production of secondary metabolites despite the relatively high abundance of biosynthetic gene clusters. This recommends that many of these clusters are completely silent or expressed at low levels under the provided culture conditions. To overcome this, epigenetic manipulation experiments can be set up.

Most of the metabolites detected from the methanolic and ethyl acetate extracts have been reported earlier to hold great importance. Here are some earlier reports on the metabolites identified from the methanolic and ethyl acetate extract of *Alanomyces manoharacharyi.* Many microorganisms have been reported to require aspartate semialdehyde to produce essential amino acids and metabolites [62]. Since 1957, N-acetyl-leucine has been available over the counter to treat vertigo [63]. Mice treated with N-acetyl-L-leucine after traumatic brain injury were capable of showing improved functional recovery, reduced neurodegeneration and neuroinflammation, and partially restored autophagy flux [64]. It has been discovered that dihydroaltersolanol C, isolated from *Stemphylium globuliferum*, found inside the plant *Juncus acutus*, can moderately inhibit *S*. *aureus* growth [65]. The medication propericiazine, sometimes known as pericyazine, is used to treat prevailing hostility, impulsivity, and aggression. In the treatment of schizophrenia, it is a standard antipsychotic medication [66]. According to Tan and Phyo [67], Mooreamide A is known to be a cannabimimetics/CNS modulatory agent. Aspergiolide isolated from cultures of the marine-derived fungus *Aspergillus glaucus* was found to selectively inhibit the proliferation of A549, HL-60, BEL-7402, and P388 cancer cell lines [68] and animal tests with mice indicated that aspergiolide inhibited tumor growth in vivo [69]. Antioxidant and potentially anti-carcinogenic α-carotene may also improve immunological function. Some epidemiological studies, but not all, found that a higher intake of α-carotene was associated with a lower occurrence of cancer and cardiovascular disease [70]. Because of its increased barrier permeability, N-(24-Hydroxytetracosanoyl) phytosphingosine is a phytoceramide essential for preserving skin health. Dryness and wrinkles result from a lower ceramide concentration in the skin. Ceramides in food may compensate for the ceramide concentration in the skin [71]. Trefely et al. [72] state that 5-Sulfosalicylic acid is an antibacterial agent. A class of related secondary metabolites known as gregatins inhibits certain facets of gram-negative bacteria’s quorum sensing [73]. According to Marino et al. [74], coscinolactam D exhibited considerable anti-inflammatory effects due to its capacity to decrease the production of PGE2 and NO. According to Kim et al. [75], photopiperazines are extremely cytotoxic metabolites that exhibit specific toxicity towards the ovarian cancer cell lines SKOV3 and U87 glioma. Thiomuracin E is a thiopeptide and potent antibiotic [76]. Osteoarthritis (OA) is commonly treated with N-acetylglucosamine (GlcNAc) [77]. Anvilone B from *Phorbas* sp. (sponge) has been isolated and reported. According to Nagai et al. [78], oscillatoxin isolated from *Moorea producens* has been shown to have diatom growth-inhibition activity against the marine diatom *Nitzschia amabilis* and cytotoxicity against the L1210 murine lymphoma cell line. Hydroquinone (HQ) is widely used in the dye industry for skin whitening, cosmetics, antioxidants, polymers, pharmaceuticals, and anticancer agents [79]. Penicoffrazin B/C has been reported to be produced from the fungus *Penicillium coffeae* isolated from *Laguncularia racemose* (Leaves, Combretaceae) [80].

The metabolite profiling of both the methanolic extract of the dried biomass and the ethyl acetate extract of the filtrate unveiled a diverse array of compounds, many of which are documented in the literature for their significant bioactivities, highlighting the potential therapeutic value and biological relevance of the compounds in these extracts. These findings not only underscore the richness of the chemical profile but also provide a promising foundation for further investigation into the specific mechanisms underlying their bioactivity. Future studies could elucidate the potential applications of these compounds in drug development and other therapeutic areas.

## 4. Conclusions

Fungi are highly appealing organisms for discovering new metabolites and biocatalysts for industrial applications. To date, around 156,000 existing species have been described from an estimated 2 to 11 million total species, with only a few thousand genomes fully sequenced and available [81]. There is an urgent need to discover new species before they become extinct. In this study, a new species of *Alanomyces* was found and its genome was sequenced and annotated. The genomic data revealed a diverse array of functional genes and pathways, suggesting a broad spectrum of biological capabilities and potential applications.

Complementing the genomic findings, LC–MS metabolite profiling of both the methanolic extract of the dried biomass and the ethyl acetate extract of the filtrate highlighted a rich and varied chemical composition. This study found that this new species of *Alanomyces* is a prolific producer of various metabolites of great industrial importance. Many of the detected metabolites are associated with known bioactivities, further emphasizing the significance of this new fungal species. The integration of genomic and metabolomic data underscores the potential of *Alanomyces manoharacharyi* as a source of novel bioactive compounds and biotechnological applications.

Overall, the findings from this study not only contribute to the understanding of fungal genomics and metabolomics but also pave the way for future research aimed at exploring the practical applications of this newly discovered species in medicine, agriculture, and industry. These findings open possibilities for targeted genome mining, such as gene knockout and the heterologous expression of genes to biosynthesize newer bioactive secondary metabolites for new drug research and development. Further research will be essential to fully elucidate the functional roles of the identified compounds and their potential therapeutic uses.

## Figures and Tables

**Table 1:** List of isolates used for constructing the phylogenetic tree of *Alanomyces manoharacharyi*.

Sr. No.	Identity	Strain	ITS	LSU
1	*Alanomyces indica*	CBS 134264	HF563622	HF563623
2	***Alanomyces manoharacharyi*** *	**NFCCI 5738**	**PP669818**	**PP669820**
3	***Alanomyces manoharacharyi*** *	**NFCCI 5739**	**PP669819**	**PP669821**
4	*Alanphillipsia aloeigena*	CPC:21286; CBS:136408	NR_137121	NG_069179
5	*Alanphillipsia aloes*	CBS 136410	MH866093	MH877631
6	*Aplosporella artocarpi*	MFLU 22-0108	ON823183	ON834371.1
7	*Aplosporella chromolaenae*	MFLUCC:17-1517	MT214340	MT214434
8	*Aplosporella hesperidica*	CBS 208.37	MH867398	
9	*Aplosporella macropycnidia*	CGMCC 3.17725	KT343648	
10	*Aplosporella papilata*	CBS 121780	EU101328	NG_070359.1
11	*Aplosporella prunicola*	CBS 121167	OM238151	KF766315
12	*Aplosporella thailandica*	MFLU 16-0615	NR_154722.1	
13	*Bagnisiella examinans*	CBS 551.66	KF766148	KF766316
14	*Barriopsis iraniana*	IRAN 1448C	NR_137030	NG_058662
15	*Barriopsis stevensiana*	CBS:174.26	MH854882.1	MH866375.1
16	*Botryobambusa fusicoccum*	MFLUCC 11-0143	JX646792	JX646809
17	*Botryosphaeria dolichospermatii*	NP1	MH491970	MH562323
18	*Botryosphaeria qingyuanensis*	CERC 2946	KX278000	MF410042
19	*Botryosphaeria ramosa*	CMW 26167	NR_151841.1	KF766333
20	*Cophinforma atrovirens*	CERC 3490	KX278009	MF410051
21	*Cophinforma mamani*	CBS 117444	KF531822	DQ377855
22	*Diplodia allocellula*	CMW 36468	JQ239397	JQ239410
23	*Diplodia seriata*	CBS 112555	KF766161	KF766327
24	*Dothiorella acacicola*	CPC 26349	KX228269	KX228320
25	*Dothiorella brevicollis*	CMW 36463	JQ239403	JQ239416
26	*Dothiorella viticola*	CBS 117010	AY905558	KX464344
27	*Endomelanconiopsis endophytica*	CBS 120397	KF766164	EU683629
28	*Endomelanconiopsis freycinetiae*	MFLUCC 17-0547	NR_158434	MG646948
29	*Endomelanconiopsis microspora*	CBS 353.97	KF766165	KF766330
30	*Eutiarosporella darliae*	CBS 118530	KX464131	KX464346
31	*Fusicladium convolvularum*	CBS 112706	AY251082	EU035428
32	*Fusicladium oleagineum*	CBS 113427	KF766166	KF766331
33	*Guignardia alliacea*	MUCC0014	AB454263	
34	*Guignardia bidwellii*	CBS 111645	EU683672	DQ377876
35	*Guignardia citricarpa*	CBS 828.97	FJ538318	KF766334
36	*Kellermania confuse*	CBS 131723	KF766174	KF766344
37	*Kellermania pseudoyuccigena*	CBS 136446; CPC:20386	MH866092	MH877630
38	*Kellermania yuccigena*	CBS 131727	KF766186	KF766356
39	*Lasiodiplodia parva*	CBS 456.78	KF766192	KF766362
40	*Lasiodiplodia theobromae*	CBS 164.96	AY640255	EU673253
41	*Macrophomina phaseolina*	CBS 162.25	KF531826	DQ377905
42	*Macrophomina tecta*	BRIP:70781	MW591684	
43	*Melanops fagicola*	MFLU 19-2862	MT185519	MT183482
44	*Melanops tulasnei*	CBS 116805	FJ824769	KF766365
45	*Mucoharknessia cortaderiae*	CPC 19974	KM108374	KM108401
46	*Neodeightonia palmicola*	MFLUCC 10-0822	NR_111550	NG_042534
47	*Neofusicoccum grevilleae*	CPC 16999	JF951137	JF951157
48	*Neofusicoccum kwambonambiense*	CBS:123639	MH863317	NG_069915
49	*Neofusicoccum parvum*	CMW 9081	KF766204	NG_042409
50	*Neoscytalidium novaehollandiae*	CBS 122071	KF766207	MH874720
51	*Phaeobotryon cupressi*	CBS 124700	MH863400	KX464538
52	*Phaeobotryon negundinis*	MFLUCC 15-0436	NR_155669	NG_069332
53	*Phyllosticta aspidistricola*	MUCC0010	AB454260	
54	*Phyllosticta capitalensis*	CBS 226.77	FJ538336	KF766377
55	*Phyllosticta citribraziliensis*	CBS 100098	OL957175	NG_069153
56	*Phyllosticta philoprina*	CBS 174.77	KF766170	KF766340
57	*Phyllosticta podocarpi*	CBS 111647	KF766217	KF766383
58	*Pileospora piceae*	DAOMC251533	MH144182	MH144186
59	*Pileospora piceae*	NB-334-4A	MH144181	MH144184
60	*Pseudofusicoccum adansoniae*	CBS 122055	KF766220	KF766386
61	*Pseudofusicoccum adansoniae*	CMW 26147	KF766220	KF766386
62	*Pseudofusicoccum kimberleyense*	CBS 122058	KF766222	MH874716
63	*Pseudofusicoccum stromaticum*	CBS 117448	KF766223	KF766389
64	*Saccharata hawaiiensis*	CBS 111787	KX464233	KX464543
65	*Saccharata kirstenboschensis*	CBS 123537	KF766225	FJ372409
66	*Saccharata leucospermi*	CBS:122694	EU552129	
67	*Saccharata proteae*	CBS 115498	KX464236	KX464546
68	*Septorioides pini-thunbergii*	CBS 473.91	MH862264	MH873946
69	*Tiarosporella madreeya*	CBS 532.76	KM108376	DQ377940
70	*Tiarosporella tritici*	CBS 118719	KC769961	DQ377941
71	*Umthunziomyces hagahagensis*	CPC 29917	KY173472	KY173561

* New species are marked in bold.

**Table 2:** List of taxa used for comparing orthologous proteins with *Alanomyces manoharacharyi* NFCCI 5738 for phylogenetic analysis and calculation of genome-scale average nucleotide identity.

Sr. No.	Organism	Strain	GenBank Assembly Accession
1	*Aplosporella prunicola*	CBS 121167	GCA_010093885.1
2	*Botryosphaeria agaves*	FJII-L1-SW-P2	GCA_022813555.1
3	*Botryosphaeria dothidea*	sdau11-99	GCA_011503125.2
4	*Botryosphaeria dothidea*	CK28	GCA_021650725.1
5	*Botryosphaeria dothidea*	GS.01s2	GCA_029169245.1
6	*Botryosphaeria kuwatsukai*	KE8637	GCA_023084525.1
7	*Botryosphaeria kuwatsukai*	PG2	GCA_004016305.1
8	*Diplodia corticola*	CBS 112549	GCA_001883845.1
9	*Diplodia intermedia*	M45-28	GCA_021495925.1
10	*Diplodia mutila*	CBS 112553	GCA_022560015.1
11	*Diplodia mutila*	KE84208	GCA_023089405.1
12	*Diplodia sapinea*	KE8364	GCA_023087385.1
13	*Diplodia sapinea*	KE8391	GCA_023087305.1
14	*Diplodia sapinea*	KE8634	GCA_023085605.1
15	*Diplodia scrobiculata*	CMW30223	GCA_001455585.1
16	*Diplodia seriata*	FDS-637	GCA_021436955.2
17	*Diplodia seriata*	M28-159	GCA_021436965.1
18	*Diplodia seriata*	F98.1	GCA_001975905.1
19	*Dothiorella sarmentorum*	KEdek	GCA_023082095.1
20	*Dothiorella sarmentorum*	KE84560	GCA_023088305.1
21	*Lasiodiplodia citricola*	KE87127	GCA_023089105.1
22	*Lasiodiplodia gonubiensis*	CBS 115812	GCA_009829795.1
23	*Lasiodiplodia iranensis*	CCTCC M2017288	GCA_030270915.1
24	*Lasiodiplodia mahajangana*	VT137	GCA_029590625.1
25	*Lasiodiplodia pseudotheobromae*	WHSB4P03s	GCA_029169085.1
26	*Lasiodiplodia pseudotheobromae*	CBS 116459	GCA_009829805.1
27	*Lasiodiplodia pseudotheobromae*	BaA	GCA_029931825.1
28	*Lasiodiplodia theobromae*	AM2As	GCA_012971845.1
29	*Lasiodiplodia theobromae*	CBS 164.96	GCA_009829825.1
30	*Lasiodiplodia theobromae*	A20-4	GCA_018153875.1
31	*Macrophomina phaseolina*	mp053	GCA_020875535.1
32	*Macrophomina phaseolina*	CBS 205.47	GCA_022204945.1
33	*Macrophomina phaseolina*	mp102	GCA_020875235.1
34	*Macrophomina phaseolina*	Al-1	GCA_008729065.1
35	*Macrophomina pseudophaseolina*	WAC 2767	GCA_022204955.1
36	*Macrophomina tecta*	BRIP 70781	GCA_024180945.1
37	*Neofusicoccum cordaticola*	CBS 123638	GCA_009830905.1
38	*Neofusicoccum cordaticola*	CBS 123634	GCA_009829355.1
39	*Neofusicoccum kwambonambiense*	CBS 123642	GCA_009829855.1
40	*Neofusicoccum kwambonambiense*	CBS 123639	GCA_009829845.1
41	*Neofusicoccum laricinum*	HLJ-QQHE-4	GCA_029906385.1
42	*Neofusicoccum laricinum*	MAFF 410183	GCA_022609205.1
43	*Neofusicoccum parvum*	DUCC19944	GCA_020912385.1
44	*Neofusicoccum parvum*	GX.1	GCA_029169195.1
45	*Neofusicoccum parvum*	SCCDJF01s	GCA_029169155.1
46	*Neofusicoccum parvum*	AH.3.1.01s	GCA_029169165.1
47	*Neofusicoccum parvum*	NSSI1	GCA_030270365.1
48	*Neofusicoccum ribis*	M1-105	GCA_021436925.1
49	*Neofusicoccum ribis*	CBS 121.26	GCA_009829435.1
50	*Neofusicoccum ribis*	CBS 115475	GCA_009829445.1
51	*Neofusicoccum umdonicola*	CBS 123644	GCA_009829365.1
52	*Neoscytalidium dimidiatum*		GCA_900092665.1
53	*Scolecobasidium constrictum*	UM 578	GCA_000611715.1
54	*Verruconis gallopava*	CBS 43764	GCA_000836295.1

**Table 3:** The peak list of spectra of *Alanomyces manoharacharyi* NFCCI 5738 indicating the protein profile (2–20 KD).

Mass-to-Charge Ratio (*m*/*z*)	Signal-to-Noise Ratio (S/N)	Relative Intensity	Intensity	Area
6159.890	6	1017	407	3152
6399.786	6	945	343	4008
6736.835	4	732	212	2959
6783.503	5	631	315	3820
6850.893	7	1115	384	4403
6863.095	5	508	278	4693
6885.070	4	1119	225	1951
13,628.300	3	2080	78.4	940
13,647.798	4	1819	93.8	1140
13,666.029	6	1736	137	2164
13,690.444	9	987	211	3387
13,703.697	11	1893	248	4071
13,725.278	10	1732	232	3510
13,744.521	7	1317	154	2397
13,763.051	6	2049	127	1642
13,781.868	5	1823	105	1228
13,799.257	5	942	115	2326
13,816.129	5	2717	111	1086
13,828.615	5	1979	109	1272
13,844.702	4	1338	96.2	1390
13,867.006	3	1868	78.7	969
13,876.685	3	1644	74.6	837

**Table 4:** Summary of assembled genomes.

Assembly	MaSurCa 4.0.5	Megahit v1.2.9	Spades v3.15.4
Contigs (≥1000 bp)	1756	946	264
Contigs (≥5000 bp)	1118	508	210
Contigs (≥10,000 bp)	850	383	189
Contigs (≥25,000 bp)	465	254	157
Contigs (≥50,000 bp)	189	175	130
Largest contig	188,634	705,157	1,303,379
Total length MB	35.11	35.11	35.55
GC (%)	50.17	50.20	50.01
N50	44,101	179,965	408,258
N75	22,880	70,317	184,668
L50	238	62	28
L75	509	140	60

**Table 5:** Putative biosynthetic gene clusters (BGCs) coding for secondary metabolites in the strain *Alanomyces manoharacharyi* NFCCI 5738.

Region	Type	From	To	Most Similar Known Cluster	Similarity
1.1	terpene	524,885	549,944	Unknown	
1.2	T1PKS	609,421	655,601	Unknown	
3.1	T1PKS	364,877	412,826	Unknown	
3.2	beta lactone	788,545	819,430	Unknown	
4.1	terpene	542,906	564,883	Unknown	
6.1	T1PKS	327,748	374,361	1,3,6,8-tetrahydroxynaphthalene	100%
9.1	NRPS	1	37,540	Unknown	
11.1	NRPS	125,072	182,954	Unknown	
13.1	NRPS-like	475,006	519,581	Unknown	
14.1	terpene	383,448	404,640	aspterric acid	100%
17.1	NRPS-like	224,629	266,991	Unknown	
21.1	fungal-RiPP-like	102,085	163,886	Unknown	
23.1	NRPS	7288	59,760	metachelin C/metachelin A/metachelin A-CE/metachelin B/dimerumic acid 11-mannoside/dimerumic acid	25%
27.1	NRPS	267,137	313,758	chaetocin	26%
31.1	T1PKS, indole	334,087	379,753	viridicatumtoxin/previridicatumtoxin/5-hydroxyanthrotainin/8-O-desmethylanthrotainin	27%
37.1	NRPS-like	270,391	314,084	Unknown	
52.1	T1PKS, NRPS	119,781	174,132	phomasetin	28%
59.1	NRPS-like	15,287	58,424	Unknown	
65.1	T1PKS	6392	51,803	(-)-Mellein	100%
67.1	T1PKS	52,155	97,544	cryptosporioptide B/cryptosporioptide A/cryptosporioptide C	15%
78.1	NRPS	52,473	99,306	metachelin C/metachelin A/metachelin A-CE/metachelin B/dimerumic acid 11-mannoside/dimerumic acid	25%
79.1	NRPS-like	42,248	85,160	Biotin	66%
92.1	NRPS	1	66,959	Unknown	
94.1	NRPS-like	58,224	93,374	Unknown	
96.1	NRPS	227	69,345	Unknown	
141.1	T1PKS	1	36,822	Patulin	26%

**Table 6:** List of the metabolites along with their classes identified from the methanolic extract of biomass and the ethyl acetate extract of the filtrate *Alanomyces manoharacharyi* NFCCI 5738 using LC–MS in positive and negative ion mode.

Compound Name	Class
Aspartate semialdehyde	Aldehydes: Amino Acid Metabolite
N-Acetyl-D-leucine	Amino Acid Derivative
PG(20:0/25:0)	Lipids: Phosphatidylglycerol Lipid
Dihydroaltersolanol and Dihydroaltersolanol C	Secondary Metabolites: tetrahydroanthraquinone
Propericiazine (oxide)	Secondary metabolites: Phenothiazine
2-trans,6-trans-farnesyl diphosphate	Lipids: Terpene Precursor
Mooreamide A	Peptide
5-Dihydroergosterol	Lipids: Sterol
Mycocerosic acid (C28)	Lipids: Fatty Acid
Aspergiolide B	Secondary metabolites: Anthraquinones
Alpha-Carotene	Lipids: Carotenoid
PE-NMe2(10:0/12:0)	Lipids: Phospholipid
DG(15:0/20:0/0:0)	Lipids: Diacylglycerol
Flavocristamide A	Peptide
N-(24-Hydroxytetracosanoyl)phytosphingosine	Lipids: Sphingolipid
5-Sulfosalicylic acid	Organic Acid
Penicoffrazin B/C	Hydroxybenzoic acid derivatives: Isocoumarins
Feruloylcholine	Phenolic Compound
7,10-dihydroxydeacetyldihydrobotrydial-1(10)-ene	Botrydial sesquiterpenoids
Gregatin G1 and Gregatin G2	Secondary metabolites: Polyketide
coscinolactam D	Secondary metabolites: Sesterterpenoids
Photopiperazine A	Cyclodipeptides: Diketopiperazines
17-hydroxy-glaciapyrrole B	Secondary metabolites: Pyrrolosesquiterpenes
clarhamnoside or LLG-1	Glycoside
GlcNAcbeta1-3Galbeta1-4(Fucalpha1-3)GlcNAcbeta1…	Carbohydrates: Oligosaccharide
Pregnane-3,6,20-triol, (3alpha,5beta,6alpha,20S)	Steroid
Chaetochiversin A	Peptide
aplyviolene	Secondary metabolites: Diterpene
Thiomuracin E	Thiopeptides: Antibiotic
Cer(d22:0/39:0)	Lipids: Ceramide
GlcNAc (N-acetylglucosamine)	Carbohydrates: Monosaccharide
CL(1′-[16:0/18:0],3′-[18:2(9Z,12Z)/20:4(5Z,8Z,1…	Lipids: Cardiolipin
Anvilone B	Secondary metabolites: Sesterterpenoids
Oscillatoxin b1/b2	Toxin
DG(16:1(9Z)/18:0/0:0)	Lipids: Diacylglycerol
TG(10:0/10:0/14:1(9Z))	Lipids: Triglyceride
DG(20:0/20:1(13Z)/0:0)	Lipids: Diacylglycerol
Maltulose	Carbohydrates: Disaccharide
Nagelamide R/Z	Peptide
Sarcohydroquinone sulfate B	Secondary metabolites: Disulfate heptaprenyl hydroquinone

## Data Availability

The data presented in this study are openly available in the NCBI GenBank database (BioProject PRJNA1114393; BioSample SAMN41484321). Other contributions presented in this study are included in the article/Supplementary Materials and further inquiries can be directed to the corresponding authors.

## Supplementary Materials

The following supporting information can be downloaded at: https://www.mdpi.com/article/10.3390/jof10110791/s1, Table S1: Genes predicted using Augustus and GeMoMa.
